# Air-Quality Assessment of On-Site Brick-Kiln Worker Housing in Bhaktapur, Nepal: Chemical Speciation of Indoor and Outdoor PM_2.5_ Pollution

**DOI:** 10.3390/ijerph16214114

**Published:** 2019-10-25

**Authors:** Steven M. Thygerson, John D. Beard, Marion J. House, Rilee L. Smith, Hunter C. Burbidge, Kathryn N. Andrus, Frank X. Weber, Ryan Chartier, James D. Johnston

**Affiliations:** 1 Department of Public Health, Brigham Young University, Provo, UT 84602, USA; john_beard@byu.edu (J.D.B.); marion.house@hotmail.com (M.J.H.); rileels@yahoo.com (R.L.S.); hburb59@gmail.com (H.C.B.); andruskathryn@gmail.com (K.N.A.); james_johnston@byu.edu (J.D.J.); 2 RTI International, Research Triangle Park, NC 27709, USA; fxw@rti.org (F.X.W.); rchartier@rti.org (R.C.)

**Keywords:** household air pollution, fine particulate matter, exposure assessment, international occupational health

## Abstract

Brick workers and their families in Nepal generally live in poorly ventilated on-site housing at the brick kiln, and may be at higher risk for non-occupational exposure to fine particulate matter air pollution and subsequent respiratory diseases due to indoor and outdoor sources. This study characterized non-occupational exposure to PM_2.5_ by comparing overall concentrations and specific chemical components of PM_2.5_ inside and outside of brick workers’ on-site housing. For all samples, the geometric mean PM_2.5_ concentration was 184.65 μg/m^3^ (95% confidence interval: 134.70, 253.12 μg/m^3^). PM_2.5_ concentrations differed by kiln number (*p* = 0.009). Kiln number was significantly associated with 16 of 29 (55%) air pollutant, temperature, or relative humidity variables. There was not a significant interaction between kiln number and location of sample for PM_2.5_ (*p* = 0.16), but there was for relative humidity (*p* = 0.02) and temperature (*p* = 0.01). Results were qualitatively similar when we repeated analyses using indoor samples only. There was no difference in the chemical makeup of indoor and outdoor PM_2.5_ in this study, suggesting that outdoor PM_2.5_ air pollution easily infiltrates into on-site brick worker housing. Outdoor and indoor PM_2.5_ concentrations found in this study far exceed recommended levels. These findings warrant future interventions targeted to this vulnerable population.

## 1. Introduction

Brick workers throughout South Asia predominantly live in impoverished conditions, while simultaneously experiencing unsafe working conditions and hazardous inhalation exposures, often starting in childhood [1,2,3,4,5,6]. Currently, there are more than 30,000 seasonal workers employed in more than 100 operating brick kilns in the Kathmandu Valley, Nepal [7,8,9]. Brick workers in Nepal suffer disproportionately from respiratory diseases [10], some of which may be directly related to silica and other hazardous occupational exposures [11]. However, it is also common in Nepal for brick workers and their families to live on-site at the kiln in rudimentary homes, where many are obliged to use cheap biomass (wood and animal dung) fuels for cooking. Consequently, in addition to unhealthy workplace exposures, many of these workers may also experience high-level exposures to household air pollutants during non-working hours [12].

Kathmandu Valley carries a natural hazard to air pollutant accumulation. The valley’s bowl-shaped topography restricts air flow in and out of the valley. This natural landscape places Kathmandu Valley at higher risk for environmental consequences, especially during the dry season from October to May. Brickmaking is a seasonal industry which coincides with the dry season, thus allowing bricks to dry in the sun. Workers are employed seasonally from December to late May each year. Brickmaking requires a migrant workforce, mostly from northern India. During the brickmaking season, workers live at the brick kiln, then return to India during the off season.

Compounding brick workers’ occupational dust and household air pollution exposures, outdoor air quality in the Kathmandu Valley has deteriorated in recent years due to increased population density and traffic [13,14]. Furthermore, most of the brick kilns in the Kathmandu Valley use coal or biomass as the primary fuel to fire their bricks. Approximately 70% of these kilns are fixed-chimney bull’s trench kilns (FCBTK), which are less fuel efficient than kilns using more modern technologies [15]. The kilns emit exhaust 24 h per day during the entire brickmaking season. Following the moment magnitude (M_w_) 7.8 Gorkha, Nepal earthquake in 2015, many toppled brick-kiln chimneys were not rebuilt to pre-earthquake heights [16,17]. Thus, kiln emissions may contribute to increased ground-level air pollution near workers’ housing.

Housing can provide a protective envelope from outdoor air pollution; however, home construction and ventilation systems heavily influence this relationship. Dockery and Spengler (1981), for instance, found that the percentage of outdoor respirable particles that infiltrate U.S. homes ranges from approximately 30%–70%. In this study, the lowest infiltration was found in tightly sealed, fully air-conditioned homes. Indoor to outdoor (I/O) PM_2.5_ and PM_10_ ratios from other studies of modern housing suggest similar infiltration rates, with ratios ranging from 0.48 to 0.81 [18,19,20]. To date, there are no studies reporting data on air pollution in Nepali brick workers’ housing, although the rustic construction of these homes suggests significant infiltration may occur. Thus, non-occupational exposures to both indoor and outdoor pollution sources may contribute significantly to the relatively high prevalence of respiratory symptoms among brick workers in Nepal [10].

Particulate matter with an aerodynamic diameter ≤ 2.5 µm (PM_2.5_) has been implicated in many of the adverse health effects associated with air pollution exposures in both adults and children [21,22,23,24,25,26]. PM_2.5_ (fine particulate matter) originates primarily from fuel combustion, including burning gasoline and diesel, coal, and biomass [21]. The health effects associated with PM_2.5_ are related to its chemical complexity and small size, which allows it to reach the alveoli by bypassing defense mechanisms in the respiratory tract. The World Health Organization (WHO) currently recommends an annual average interim target (IT-1) of 35 µg/m^3^ for indoor PM_2.5_ levels [27]. By comparison, cooking and heating indoors with biomass fuels can result in household air pollution levels that are orders of magnitude higher than current WHO recommendations [12,28]. Several studies also suggest that the specific chemical components of PM_2.5_, in addition to the overall concentration, are responsible for its adverse health effects [29,30,31]. Understanding both the concentration and chemical composition of PM_2.5_ in brick workers’ homes may be important for guiding future interventions to reduce exposures in this population of workers. There is relatively little data on brick workers’ exposures and health in Nepal. Thus, the purpose of this cross-sectional study is to determine: (A) What is the magnitude of indoor and outdoor PM_2.5_ exposures for Nepali brick-kiln workers living on-site at the brick kiln; and (B) Does ambient air pollution infiltrate into on-site brick-kiln workers’ housing? Additionally, we analyzed the chemical composition of PM_2.5_, and made comparisons between indoor and outdoor samples.

## 2. Materials and Methods

Brick workers’ homes (N = 16) were selected by convenience sampling from four different kilns located in Bhaktapur, Nepal for this cross-section study. Four homes from each kiln were sampled during May 2018. All homes were located on the brick-kiln property, within approximately 100 m of the kiln. Homes were classified as either fire master or worker (non-fire master) homes. At each kiln, we sampled two fire master homes and two worker homes. Fire master homes were classified separately because in three of the four kilns the fire master home was located directly on the kiln, whereas the worker homes were located adjacent to the kiln. [Institution name removed for blind peer review] University’s Institutional Review Board determined that this study was exempt because it did not meet the definition of human subjects research outlined in 45 CFR 46 [32].

**Figure 1:**
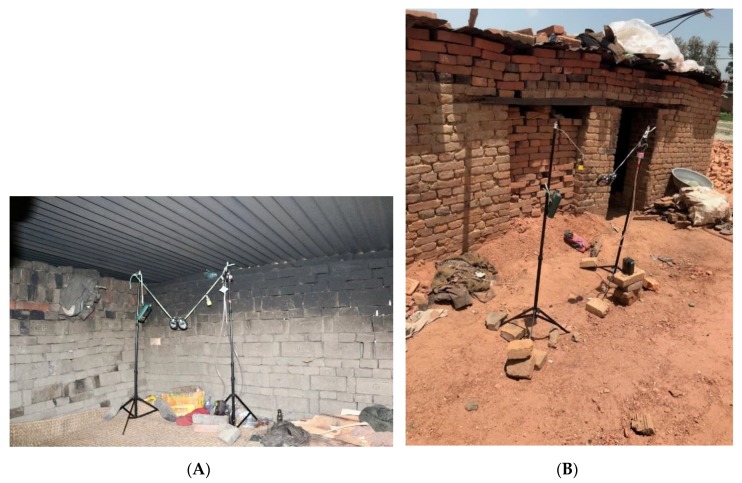
Equipment setup for indoor and outdoor sampling; (**A**): indoor air sampling setup; (**B**): outdoor air sampling setup.

At each of the four brick-kiln sites that we visited we collected indoor and outdoor measures of fine particulate matter ≤ 2.5 μm (PM_2.5_), air temperature (°C), and percent relative humidity (RH). Monitoring equipment was set up and started in the morning, and picked up in the afternoon on the same day. Many of the brick-kiln workers’ homes had low roofs (approximately 1.5 m high). Therefore, monitoring equipment was placed approximately 1.1–1.2 m off the ground, which we estimated to be the breathing zone height for occupants crouching or kneeling in the home. Monitoring equipment was suspended on a string between two tripods, and located against one wall in the home. We used the same monitoring set up for both indoor and outdoor samples. Outdoor samples were located outside within five meters of the home. In some cases, the outdoor samples were placed next to the home, and occasionally under simple cover, to protect the equipment from rain. Figure 1 shows the equipment setup for indoor and outdoor sampling.

### 2.1. PM_2.5_ Measurement

To determine the concentration of PM_2.5_, we used SKC AirLite sampling pumps (SKC, Inc., Eighty Four, PA) to draw air at 2.0 L/min through personal exposure monitors (PEM; SKC, Inc.) with a cut point of 2.5µm. We applied vacuum grease to the impaction plates prior to sampling to limit particle bounce. Particulate matter was collected on pre-weighed 37 mm polytetrafluoroethylene (PTFE) filters with a 2.0 µm pore size (SKC, Inc.). PTFE filters were pre- and post-weighed on a Mettler Toledo XS3DU (Mettler Toledo, Columbus, OH) microbalance (detection limit mass: one μg with 99.9% accuracy if measuring at least one mg of weight; the average pre-weight of the PTFE filters we used was 56.47 mg). Field blanks (n = 5) were treated similar to the other sampling filters, although no air was pulled through them. Prior to sampling each day, new batteries were placed in the SKC AirLite pumps. Pumps were pre- and post-calibrated with the sampling train in place using a Dry Cal Defender 510 volumetric flow calibrator (Mesa Labs, Butler, NJ). All but three of the 32 post-calibrations were within ±5% of the 2.0 L/min target flow rate.

### 2.2. PM_2.5_ Carbon Analysis

Following gravimetric analysis, all sample filters were shipped to RTI (RTI International, Research Triangle Park, NC, USA) for optical analysis. Optical analysis was performed using a modified version of RTI’s integrating sphere optical filter transmittance method [33,34]. This technique measures optical transmittance through the filter and deposited sample at seven discrete wavelengths ranging from blue (430 nm) to near-infrared (940 nm). The wavelength-dependent change in transmission thru the filter from pre- to post-sampling is used to estimate the species-specific mass loading collected during sampling. Black carbon (BC) absorbs strongly at all wavelengths including near-infrared, while brown carbon (BrC) absorbs more light at shorter wavelengths and less in the near-infrared. The mass fraction of each species was calculated using an empirically-derived algorithm that iteratively adjusts the mass fraction of each species to minimize the difference between the measured and modeled optical properties of the collected particulate matter.

### 2.3. PM_2.5_ Elemental Composition Measurement

RTI, International (RTI International, Research Triangle Park, NC, USA) performed the analysis of the 37mm filters for 33 elements (Supplemental Table S1) following the IO3.3 compendium method [35], modified for use using the Thermo (Thermo Fisher Scientific, Waltham, MA, USA) ARL energy-dispersive X-ray fluorescence (EDXRF) instrument. The X-ray excitation energies, filters, and counting times were optimized to achieve the best signal while minimizing the impacts of overlapping energy lines. A camera system within the instrument chamber was used to ensure the beam was focused on the exposed area of the filter to accurately quantify the elements of concern. The EDXRF used in this study was equipped with a silicon drift detector (SDD). This instrument configuration was used because it can produce enough spectral counts to fully quantify each element, while collimating the beam. A commercially purchased (Micromatter Technologies, Inc., Surrey, British Columbia, CA) thin-film standard was run as a sample every 10th analysis to demonstrate recovery and assess instrument drift.

### 2.4. Air Temperature and Relative Humidity

Air temperature (°C) and RH (%) were monitored using Extech (Extech Instruments, Nashua, NH) Model SD500 Datalogging Hygro-Thermometers (TRH meters). Prior to the study, we identified and labeled eight (TRH 1–8) similar meters out of 20 potential meters by assessing accuracy and precision in a controlled lab environment. Additionally, two more of the 20 potential meters were chosen and labeled as replacements in case of meter failure (BU1–2). Eight of the meters were used daily at each kiln; one indoor and one outdoor for each home. For each meter prior to each sampling period, data collection intervals were reset to 60 s, new batteries were installed, and the SD card was cleared. The meters were attached to the suspension string and sampling was initiated at the same time as the other instrumentation. Readings were taken every 60 s throughout the duration of the sampling period. After sampling, the data was transferred to computer storage as Excel spreadsheets via the SD card. After sampling kiln three, TRH 2 failed to reset and BU1 was used as a replacement in the next sampling period.

### 2.5. Housing Questionnaire

With the help of an interpreter, we interviewed an adult resident of each home in Nepali, using a Nepali version of a 14-item housing questionnaire. The purpose of the questionnaire was to identify home characteristics that may be related to PM_2.5_ concentrations, thereby suggesting directions for future intervention research. Questionnaires items included size of home, time lived in home, total number of people and number of children living in the home, any previous water damage, whether residents cooked in the home, the type of fuel used, presence or absence of a chimney or other ventilation system, heating and lighting sources, number of smokers who lived in home, number of smokers who lived in home who regularly smoked inside, and number of indoor dog, cat, or rodent pets.

### 2.6. Statistical Analyses

We conducted all analyses using SAS version 9.4 (SAS Institute Inc., Cary, NC). For PM_2.5_ and PM_2.5_ chemical components, we determined whether any of the field blank samples had measurements above detection limits. PM_2.5_, PM_2.5_ bromine, PM_2.5_ chromium, PM_2.5_ iron, PM_2.5_ magnesium, and PM_2.5_ sulfur had at least one field blank sample that had measurements above detection limits, so we used the maximum field blank measurement for analyses instead of the detection limit. For temperature and RH, we calculated arithmetic means for each sample using simple linear regression models. For home characteristics, we calculated frequencies and percentages for categorical variables, and arithmetic means, standard deviations, minimums, first quartiles, medians, third quartiles, and maximums for continuous variables. Virtually all air pollutant concentrations, temperature, and RH distributions were right skewed, so we calculated geometric means (GM) and 95% confidence intervals (CI) using simple linear or Tobit regression models. This was done by using natural logarithm transformations of air pollutant, temperature, and RH measurements as dependent variables and exponentiating the regression coefficients. We used simple Tobit regression models to complete these calculations when some measurements were below detection limits. We also calculated minimums and maximums for each air pollutant, temperature, or RH variable.

If more than 10% of samples for a particular air pollutant, temperature, or RH variable had measurements above detection limits (or the maximum field blank sample measurement), then we estimated unadjusted associations between home characteristics and that air pollutant concentration, temperature, or RH variable using simple exact unconditional logistic, linear, or Tobit regression models. We used exact unconditional logistic regression models when greater than 10% to 30% of samples for a particular air-pollutant, temperature, or RH variable had measurements above detection limits, Tobit regression models when greater than 30% to 99% of samples for a particular air pollutant, temperature, or RH variable had measurements above detection limits, and linear regression models when 100% of samples for a particular air pollutant, temperature, or RH variable had measurements above detection limits [36,37]. We used natural logarithm transformed air pollutant concentrations and temperature and RH variable measurements as dependent variables in the linear and Tobit regression models. For linear and Tobit regression model analyses and when appropriate, we evaluated pairwise differences in air pollutant concentrations or temperature RH measurements among categories of home characteristics and used the Tukey (linear regression models) or Tukey–Kramer (Tobit regression models) method to adjust *p*-values for multiple comparisons. We also evaluated interactions between kiln number and location of sample for each air pollutant or temperature or RH variable. As a secondary analysis, we again estimated unadjusted associations between home characteristics and air pollutant concentrations, temperature, and RH measurements using indoor samples only.

## 3. Results

The average home size was 7.64 m^2^ (82.16 ft^2^) and individuals had lived in the homes a median of 5.50 months (Table 1). A median of 4.00 people lived in the houses, with a median occupant density of 65.15 residents/100 m^2^. Thirty-six percent of households reported having 1–3 children aged 0–18 years old living in the house and 31% reported having 1–3 children aged 0–6 years old living in the house. None of the households reported any serious water damage, and 100% of residents cooked inside the home. Sixty percent of households reported using wood as the primary fuel for cooking, but no household reported having a stove or other cooking source vented to the outdoors with a chimney. Ninety-three percent of homes had a heating source in the home and 67% reported the type of heating source was electricity. Ninety-three percent of homes had a non-electric light source in the home and 50% reported the type of non-electric light source was candles only. Sixty-seven percent of homes had at least one smoker, and 25% of homes had 3–4 smokers. Thirty-three percent of homes that had smokers living in them had 2–4 smokers who regularly smoked inside the home. None of the households reported having dogs, cats, or rodent pets living in the home. All of the homes in this study were of similar construction. Homes were built with fired (red) or unfired (green) bricks. Roofs were made by suspending bamboo poles across the roof opening, and covering this with sheets of tin. Tin roofing was held down by bricks or other heavy items. All homes used a natural fiber carpet to cover the door opening, and no homes in this study had windows.

The GM for all PM_2.5_ samples combined (indoor and outdoor) was 184.65 μg/m^3^ (Table 2). There was not a significant difference between indoor (182.80 μg/m^3^) and outdoor (186.52 μg/m^3^) PM_2.5_ concentrations (*p* = 0.06), but there was a significant difference in the GM PM_2.5_ air concentrations among kilns (*p* = 0.009; Supplemental Table S2). Tests of pairwise differences indicated that the significant difference was attributed to kilns three (GM = 364.61 μg/m^3^) and four (GM = 91.06 μg/m^3^, *p* = 0.005). No other home characteristic, including type of home (fire master vs. worker: *p* = 0.14) and location of sample (indoor vs. outdoor: *p* = 0.95), was significantly associated with PM_2.5_ air concentrations.

Less than or equal to 10% of samples had measurements above detection limits for 11 PM_2.5_ chemical components, but greater than 10% of samples had measurements above detection limits for PM_2.5_ and 24 PM_2.5_ chemical components (Supplemental Tables S1 and S2). The GMs for the 24 PM_2.5_ chemical components ranged from 0.0041 μg/m^3^ for PM_2.5_ strontium to 15.19 μg/m^3^ for PM_2.5_ black carbon (Table 2). The GM temperature was 26.56 °C and the GM RH was 40.51% (Table 2).

Kiln number was significantly associated with 13 of 24 (54%) PM_2.5_ chemical components; kiln three had the highest concentration for 11 of these associations and kiln four had the lowest concentration for 11 (Supplemental Tables S2–S10). Location of sample was significantly associated with one (4%) PM_2.5_ chemical component: indoor samples had a higher PM_2.5_ chlorine concentration than outdoor samples (0.34 vs. 0.14 μg/m^3^, *p* = 0.05). Size of house was significantly associated with one (4%) PM_2.5_ chemical component: a 50 ft^2^ increase in size of house was associated with a 558% increase in the odds of PM_2.5_ sodium concentrations above the detection limit (*p* = 0.03; for a one m^2^ increase in size of house, the odds ratio was 1.50, *p* = 0.03). How long lived in house was significantly associated with 12 (50%) PM_2.5_ chemical components and 11 of these associations were positive. How many people live in house was significantly associated with two (8%) PM_2.5_ chemical components and both of these associations were inverse. Occupant density was significantly associated with two (8%) PM_2.5_ chemical components and both of these associations were inverse. Primary fuel used for cooking was significantly associated with two (8%) PM_2.5_ chemical components; gas only had the lowest concentration and wood only had the highest concentration for both of these associations. Type of heating source in the home was significantly associated with four (17%) PM_2.5_ chemical components and electricity had lower concentrations than other or none for all four of these associations. Any smokers living in the home was significantly associated with 12 (50%) PM_2.5_ chemical components and any smokers living in the home had lower concentrations than no smokers living in the home for all 12 of these associations. How many smokers living in the home was significantly associated with 10 (42%) PM_2.5_ chemical components; zero had the highest concentration for nine of these associations and one to two had the lowest concentration for eight. How many smokers living in the home regularly smoke inside the home was significantly associated with one (4%) PM_2.5_ chemical component: zero to one had a higher PM_2.5_ sodium concentration than two to four (2.38 vs. 1.15 μg/m^3^, *p* = 0.007). Type of home, how many children 0–18 years-old live in house, how many children under six years-old live in house, and type of non-electric light source in the home were not significantly associated with any PM_2.5_ chemical components.

There was a significant difference in GM RH among kilns (*p* < 0.0001) (Supplemental Table S10). Tests of pairwise differences indicated that only kilns three (GM = 53.69%) and four (GM = 65.13%) had GM RH percentages that were not significantly different (*p* = 0.12).

There was a significant inverse association between number of people living in the house and RH (exp(β) = 0.87; 95% CI: 0.80, 0.93; *p* = 0.0005). Primary fuel used for cooking was significantly associated with RH (*p* = 0.02). Tests of pairwise differences indicated a significant difference between gas only (GM = 54.84%) and other (coal and wood, gas and wood) (GM = 26.87%, *p* = 0.01). RH was significantly associated with type of heating source in the home (electricity: GM = 52.92%; other (lightbulb, line cable) or none: GM = 26.20%, *p* < 0.0001). Type of non-electric light source in the home was significantly associated with RH (*p* < 0.0001). Tests of pairwise differences indicated significant differences between candle (GM = 51.18%) and generator (GM = 22.56%, *p* < 0.0001) and generator and other (candle and torch, fuel) or none (GM = 51.45%, *p* < 0.0001). RH was significantly associated with number of smokers living in the home who regularly smoke inside the home (0–1: GM = 55.10%; 2–4: GM = 36.12%, *p* = 0.03).

There was a significant difference in GM temperature among kilns (*p* < 0.0001) (Supplemental Table S10). Tests of pairwise differences indicated that only kilns two (GM = 26.50 °C) and three (GM = 27.03 °C) had GM temperatures that were not significantly different (*p* = 0.97). There was a significant positive association between temperature and number of people living in the house (exp(β) = 1.03; 95% CI: 1.00, 1.06; *p* = 0.04). Temperature was significantly associated with primary fuel used for cooking (*p* = 0.01). Tests of pairwise differences indicated significant differences between gas only (GM = 22.62 °C) and wood only (GM = 27.28 °C, *p* = 0.02) and gas only and other (coal and wood, gas and wood) (GM = 29.11 °C, *p* = 0.01). Type of heating source in the home was significantly associated with temperature (electricity: GM = 24.12 °C; other (lightbulb, line cable) or none: GM = 30.26 °C, *p* = 0.0004). Temperature was significantly associated with type of non-electric light source in the home (*p* = 0.0003). Tests of pairwise differences indicated significant differences between candle (GM = 24.65 °C) and generator (GM = 31.51 °C, *p* = 0.0003) and generator and other (candle and torch, fuel) or none (GM = 24.70 °C, *p* = 0.003). The presence of smokers living in the home was significantly associated with temperature (No: GM = 29.35 °C; Yes: GM = 25.26 °C, *p* = 0.01).

**Figure 2:**
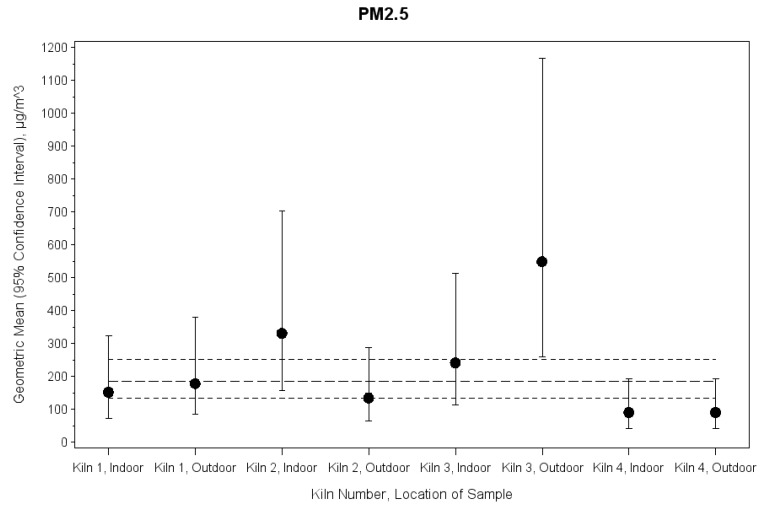
Interaction between kiln number and location of sample (p for interaction = 0.16) and PM_2.5_ measured at homes at brick kilns in Bhaktapur, Nepal, May 2018 (actual values of geometric means and 95% confidence intervals are included in Table 3). The large dashed line shows the overall geometric mean and the small dashed lines show the 95% confidence interval for the overall geometric mean. Abbreviations: PM_2.5_, particulate matter with an aerodynamic diameter less than 2.5 μm.

Interactions between kiln number and location of sample were statistically significant for PM_2.5_ lead (*p* = 0.03), RH (*p* = 0.02), and temperature (*p* = 0.01), but not for PM_2.5_ (*p* = 0.16) or any other PM_2.5_ chemical component (Figure 2, Table 3).

Although there were fewer significant associations when we estimated unadjusted associations between home characteristics and air pollutant concentrations, temperature, or RH using indoor samples only, perhaps because of smaller sample sizes and reduced statistical power, results were generally similar (not shown). The two home characteristics that experienced the largest decreases in numbers of significant associations were how long lived in house (zero significant associations using indoor samples only) and how many smokers living in the home (two significant associations using indoor samples only). The associations that were significant when we used indoor samples only were almost always the same associations that were significant when we used all samples and the significant associations were almost always in the same directions.

## 4. Discussion

To the best of our knowledge, this is the first study to characterize indoor air pollution in on-site brick workers’ homes in Nepal. In order to put this study in perspective, it might be helpful to discuss some limitations to the work first. We had limited access to the kilns for this study, and were obliged to collect our samples during daytime hours. Thus, our results must be considered in light of the partial-day exposures that we measured, when most of the men and many of the women were at work, and when there was little cooking taking place in the homes. We arrived at the kilns after the morning meal had been prepared, and we collected our equipment in the late afternoon of the same day before the evening meal was prepared or when candles were used for light in the homes, or home occupants started smoking indoors. Thus, our results most likely significantly underestimate the indoor PM_2.5_ exposures experienced by brick workers and their families, especially during evening and morning hours. However, even under these conservative sampling conditions, the geometric mean indoor PM_2.5_ concentration across the 16 homes sampled in this study was 182.80 μg/m^3^, which is approximately 5.2 times greater than the WHO recommended annual average of 35 µg/m^3^ for indoor PM_2.5_ levels [27]. Our study was limited to sampling at 16 homes. However, these homes were at four separate kilns located in different sections of Bhaktapur, Nepal. Indoor and outdoor samples resulted in the analysis of 32 samples for multiple contaminants.

We also documented several characteristics of brick workers’ homes that may be related to high indoor PM_2.5_ exposures. Perhaps most importantly, the on-site housing was small, averaging less than 8.0 m^2^, with significant overcrowding. The mean occupant density was approximately 75.0 persons/100m^2^, far higher than that found in low-income housing in developed countries. The small size and high occupant densities in brick workers’ on-site housing may be important when considering the various combustion sources in the homes, and how indoor pollution may concentrate in these small, poorly ventilated structures. Of note, not a single home in our study had a stove or other cooking source vented to the outdoors with a chimney. Additionally, 60% of homes in this study used an open wood fire in the home as the primary cooking source. Household air pollution from indoor cooking with biomass fuels is a major public health issue globally [27], and our findings suggest this practice is common among Nepali brick workers living in on-site housing. We also found high rates of smoking, and smoking indoors among home occupants. For half of the homes in this study, candles were used as the only means of light, which may also contribute to the overall indoor PM_2.5_ burden. Based on our findings, we suggest that household air pollution from indoor cooking with biomass fuels, in combination with other combustion sources such as smoking and use of candles, may be a serious and pervasive problem in on-site brick worker housing in Nepal. However, due to limitations in this study we were not able to show this empirically. These results are informative, however, and can be used to guide future studies to more fully characterize indoor pollution exposures in this population of workers.

Our findings from the outdoor samples are also of interest. Many of the brick kilns in the Kathmandu Valley are FCBTK, which contribute significantly to outdoor air pollution [9]. Incomplete combustion in FCBTK results in higher emission values when compared to zig-zag kilns, vertical shaft kilns or Hoffman kilns. The average value of PM emissions are within the regulatory limit, however, some of the kilns emit higher PM [38]. Due to the shorter chimney heights of the FCBTK following the 2015 earthquake, and due to increased traffic and population density, air pollution levels in the valley have increased over time. For example, two previous reports show outdoor PM_2.5_ concentrations in the Kathmandu Valley at 125 and 140 ug/m^3^ [13,14]. Our findings add to these previous studies. We found an average outdoor PM_2.5_ concentration across the four brick kilns of 186.52 ug/m^3^. This level far exceeds the WHO recommended annual and 24 hr mean levels of 10 ug/m^3^ and 25 ug/m^3^, respectively [39]. Much of this pollution likely originates from the kilns themselves. In addition, many of the kilns in Bhaktapur are located along the Araniko Highway. This highway, built in the 1960s, is the major thoroughfare from Kathmandu through Bhaktapur and into the eastern regions of Nepal and eventually to the Nepal/China border. Thus, we suspect that a high percentage of the PM_2.5_ pollution measured in this study originated from vehicle exhaust on the highway. One factor that may contribute to elevated levels of outdoor particulate matter is the relative low wind speeds in the Kathmandu Valley. The brickmaking season is during the windiest part of the year. Yet wind speeds average less than 8 km per hour. There is no predominant wind direction. However, wind rarely comes from the east. The kilns in this study were located 10 km east of Kathmandu [40].

Mean indoor PM_2.5_ concentration and PM_2.5_ components, with the exception of chlorine, did not differ significantly from outdoor samples. This was not surprising, considering that we did not sample during prime cooking hours for morning and evening meals. We suggest that the indoor levels measured in this study were likely due to infiltration of outdoor air pollution into homes. Typical brick worker homes have dirt floors, are small, and have low roofs and high occupant densities. The walls are generally constructed with either red (fired) or green (unfired) bricks from the kiln, and are often not mortared together, leaving significant gaps between bricks. The roof of the home is most often constructed from tin sheets placed over bamboo poles, leaving gaps between the tops of walls and the roof. Natural fiber mats are used to cover doorway openings. Thus, gaps in the building materials allow for infiltration of outdoor air pollution. We are not sure what might account for the higher levels of chlorine found indoors vs. outdoors. In comparison to modern homes in westernized countries, the I/O ratios found in this study were quite high. For PM_2.5_, the I/O ratio in the on-site brick workers’ housing was 0.98. By comparison, I/O particulate ratios in modern housing in westernized countries ranges from 0.48 to 0.81 [18,19,20]. When looking at individual PM_2.5_ constituents, we found similarly high ratios. These results suggest a high percentage of outdoor air pollution infiltrates into brick workers’ homes.

Several relatively low-cost measures may help to significantly reduce infiltration of outdoor air pollution into brick workers’ homes. First, many of the homes were constructed with bricks that were not mortared together. Infiltration of outdoor air pollution can be significantly limited by sealing gaps between the bricks and between the walls and the roof. Home doorways may also be a significant source of outdoor pollution infiltration since the natural fiber carpets that cover the doorway are not tight-fitting. Using a rigid, tight-fitting door may provide a better seal. However, sealing gaps in the homes may exacerbate indoor pollution levels from cooking, smoking, and burning candles. Low-cost chimney stoves have been shown to significantly reduce indoor emissions in similar rustic housing [41]. Smoking cessation programs may help reduce indoor pollutants, as well as simple electric light bulbs rather than candles.

There was also no difference in indoor PM_2.5_ concentration or components based on type of home. Firemans’ homes are located directly on or next to the kiln trench. Other workers’ homes are located off the kiln trench and up to 100 m away from the kiln itself. However, there was no significant difference between the air contaminant concentrations in these two types of homes. Home construction, size, and occupant density were similar for both types of homes. Thus, home location on the kiln property did not seem to affect indoor air contaminant concentrations. Twenty-four PM_2.5_ chemical components we measured had greater than 10% of samples with measurements above detection limits. Compared to arithmetic mean concentrations reported in California [31,42], the GM concentrations we measured were higher for 11 PM_2.5_ chemical components (aluminum, calcium, chlorine, iron, potassium, manganese, lead, sulfur, silicon, titanium, zinc), similar for three (bromine, nickel, vanadium), and lower for one (copper). Compared to arithmetic mean concentrations reported in China [43,44,45,46], the GM concentrations we measured were higher for three PM_2.5_ chemical components (black carbon, iron, titanium), similar for three (calcium, magnesium, potassium), and lower for seven (arsenic, bromine, chlorine, lead, manganese, sodium, zinc). Concentrations may have been higher for most PM_2.5_ chemical components we measured relative to those measured in California because the concentration of total PM_2.5_ we measured, 184.65 μg/m^3^, was about nine times as high as that measured in California: 19.3 μg/m^3^ [31,42]. However, concentrations of total PM_2.5_ measured in China, 55 μg/m^3^ in one study and 115 μg/m^3^ in another [43,46], were also lower than what we measured, which may mean the chemical composition and sources of the PM_2.5_ we measured were different from those of the PM_2.5_ measured in China. Future research in Nepal should determine the sources of PM_2.5_ air pollution for brick-kiln workers and their families. The brick industry should continue to investigate the latest production technologies to reduce emissions and improve worker and community health.

## 5. Conclusions

In conclusion, outdoor and indoor PM_2.5_ concentrations found at homes of brick-kiln workers and their families at brick kilns in Bhaktapur, Nepal, far exceed recommended levels. There was also a wide range of PM_2.5_ chemical components inside and outside brick workers’ homes. There was no difference in the concentrations or chemical makeup of indoor and outdoor PM_2.5_ in this study, suggesting that outdoor PM_2.5_ air pollution easily infiltrates into on-site brick-worker housing. These findings warrant future interventions to reduce PM_2.5_ concentrations among the vulnerable population of brick-kiln workers and their families in Nepal.

## Figures and Tables

**Table 1:** Characteristics of on-site homes at brick kilns in Bhaktapur, Nepal, May 2018.

Characteristic	Homes, n (%)	Missing, n	Mean	SD	Min	Q1	Median	Q3	Max
Total	16 (100)								
Kiln number									
1	4 (25)								
2	4 (25)								
3	4 (25)								
4	4 (25)								
Fire master home	8 (50)								
Size of house, feet^2^		2	82.16	37.09	20.00	52.00	84.00	112.00	150.00
Size of house, m^2^		2	7.64	3.42	1.90	4.80	7.80	10.40	13.90
How long lived in house, months		2	8.82	8.04	4.00	5.00	5.50	7.00	30.00
How many people live in house		4	4.71	2.24	2.00	3.00	4.00	6.00	9.00
Occupant density, residents/100 m^2^		4	74.66	48.91	19.23	43.81	65.15	89.28	210.53
How many children 0–18 years-old live in house		2							
0	9 (64)								
1–3	5 (36)								
How many children under 6 years-old live in house		3							
0	9 (69)								
1–3	4 (31)								
Any serious water damage to the home	0 (0)	1							
Cook inside the home	15 (100)	1							
Primary fuel used for cooking		1							
Gas only	3 (20)								
Wood only	9 (60)								
Other ^A^	3 (20)								
Stove or other cooking source vented to the outdoors with a chimney	0 (0)	2							
Heating source in the home	14 (93)	1							
Type of heating source in the home		4							
Electricity	8 (67)								
Other ^B^ or none	4 (33)								
Non-electric light source in the home	14 (93)	1							
Type of non-electric light source in the home		2							
Candle only	7 (50)								
Generator	4 (29)								
Other ^C^ or none	3 (21)								
Any smokers living in the home	10 (67)	1							
How many smokers living in the home		4							
0	5 (42)								
1–2	4 (33)								
3–4	3 (25)								
How many smokers living in the home regularly smoke inside the home		1							
0–1	6 (67)								
2–4	3 (33)								
Dogs currently living in home	0 (0)	1							
Cats currently living in home	0 (0)	1							
Rodent pets	0 (0)	1							

Abbreviations: Max, maximum; Min, minimum; Q1, first quartile 1; Q3, third quartile; SD, standard deviation; ^A^ Includes coal and wood, gas and wood; ^B^ Includes lightbulb, line cable; ^C^ Includes candle and torch, fuel.

**Table 2:** Summary statistics for air pollutants, relative humidity, and temperature measured at on-site homes at brick kilns in Bhaktapur, Nepal, May 2018.

Air Pollutant or Weather Variable ^A^	Total Samples						
	Missing, n	Below DL ^B^, n (%)	Above DL ^B^				
			n (%)	GM ^C^	95% CI ^C^	Min ^D^	Max ^D^
PM_2.5_, μg/m^3^		0 (0) ^E^	32 (100) ^E^	184.65	134.70, 253.12	53.30	2438.10
PM_2.5_ aluminum, μg/m^3^		0 (0)	32 (100)	3.53	2.24, 5.55	0.33	143.23
PM_2.5_ black carbon, μg/m^3^		0 (0)	32 (100)	15.19	10.58, 21.80	5.21	289.30
PM_2.5_ barium, μg/m^3^		8 (25)	24 (75)	0.21 ^F^	0.14, 0.32 ^F^	0.13	7.59
PM_2.5_ brown carbon, μg/m^3^		12 (38)	20 (63)	0.76 ^F^	0.24, 2.40 ^F^	0.27	531.50
PM_2.5_ bromine, μg/m^3^		28 (88) ^E^	4 (13) ^E^	0.0052 ^F^	0.0012, 0.024 ^F^	0.027	0.11
PM_2.5_ caesium, μg/m^3^		15 (47)	17 (53)	0.10 ^F^	0.067, 0.16 ^F^	0.10	2.29
PM_2.5_ calcium, μg/m^3^		0 (0)	32 (100)	1.03	0.71, 1.51	0.14	23.92
PM_2.5_ chlorine, μg/m^3^		4 (13)	28 (88)	0.22 ^F^	0.14, 0.35 ^F^	0.074	8.62
PM_2.5_ chromium, μg/m^3^		22 (69) ^E^	10 (31) ^E^	0.070 ^F^	0.050, 0.098 ^F^	0.088	0.34
PM_2.5_ cobalt, μg/m^3^		16 (50)	16 (50)	0.012 ^F^	0.0064, 0.021 ^F^	0.014	0.58
PM_2.5_ iron, μg/m^3^		0 (0) ^E^	32 (100) ^E^	3.10	1.98, 4.87	0.29	118.29
PM_2.5_ lead, μg/m^3^		21 (66)	11 (34)	0.015 ^F^	0.0081, 0.028 ^F^	0.026	0.15
PM_2.5_ magnesium, μg/m^3^		22 (69) ^E^	10 (31) ^E^	0.097 ^F^	0.071, 0.13 ^F^	0.14	0.25
PM_2.5_ manganese, μg/m^3^		9 (28)	23 (72)	0.035 ^F^	0.022, 0.054 ^F^	0.021	1.05
PM_2.5_ nickel, μg/m^3^		23 (72)	9 (28)	0.0042 ^F^	0.0017, 0.011 ^F^	0.013	0.28
PM_2.5_ potassium, μg/m^3^		0 (0)	32 (100)	1.90	1.28, 2.81	0.26	35.64
PM_2.5_ rubidium, μg/m^3^		21 (66)	11 (34)	0.010 ^F^	0.0048, 0.021 ^F^	0.020	0.41
PM_2.5_ silicon, μg/m^3^		0 (0)	32 (100)	8.00	5.29, 12.08	0.85	222.48
PM_2.5_ sodium, μg/m^3^		27 (84)	5 (16)	0.18 ^F^	0.12, 0.28 ^F^	0.29	0.45
PM_2.5_ strontium, μg/m^3^		24 (75)	8 (25)	0.0041 ^F^	0.00076, 0.023 ^F^	0.028	1.36
PM_2.5_ sulfur, μg/m^3^		0 (0) ^E^	32 (100) ^E^	2.20	1.79, 2.70	0.86	12.27
PM_2.5_ titanium, μg/m^3^		3 (9)	29 (91)	0.35 ^F^	0.21, 0.57 ^F^	0.055	20.30
PM_2.5_ vanadium, μg/m^3^		27 (84)	5 (16)	0.0045 ^F^	0.00062, 0.033 ^F^	0.035	0.68
PM_2.5_ zinc, μg/m^3^		5 (16)	27 (84)	0.046 ^F^	0.031, 0.068 ^F^	0.016	0.67
Relative humidity, %	1	NA	31 (100)	40.51	34.40, 47.70	14.87	69.22
Temperature, °C	1	NA	31 (100)	26.56	25.13, 28.08	20.35	38.53

Abbreviations: CI, confidence interval; DL, detection limit; GM, geometric mean; Max, maximum; Min, minimum; NA, not applicable; PM_2.5_, particulate matter with an aerodynamic diameter less than 2.5 μm; ^A^ Eleven air pollutants measured above detection limits in less than 10% of samples. The names, detection limits, and summary statistics for these air pollutants are included in Supplemental Table S1; ^B^ Detection limits are included in Supplemental Table S1; ^C^ Estimated via linear regression models of the natural logarithm transformed values; ^D^ Calculated from samples that had values above detection limits; ^E^ Maximum mass and concentration ranges from blank samples that measured above detection limits were used instead of detection limit masses and concentration ranges; ^F^ Estimated via Tobit regression models of the natural logarithm transformed values.

**Table 3:** Interactions between kiln number and location of sample and air pollutants, relative humidity, and temperature measured at on-site homes at brick kilns in Bhaktapur, Nepal, May 2018.

Air Pollutant or Weather Variable ^A^	Kiln Number by Location of Sample								*p* for Interaction ^C^
	Kiln 1, Indoor	Kiln 1, Outdoor	Kiln 2, Indoor	Kiln 2, Outdoor	Kiln 3, Indoor	Kiln 3, Outdoor	Kiln 4, Indoor	Kiln 4, Outdoor	
	GM (95% CI) ^B^	GM (95% CI) ^B^	GM (95% CI) ^B^	GM (95% CI) ^B^	GM (95% CI) ^B^	GM (95% CI) ^B^	GM (95% CI) ^B^	GM (95% CI) ^B^	
PM_2.5_, μg/m^3^	152.58 (71.87, 323.94)	178.90 (84.27, 379.83)	331.46 (156.12, 703.72)	135.51 (63.83, 287.70)	241.99 (113.98, 513.76)	549.38 (258.76, 1166.37)	91.25 (42.98, 193.72)	90.87 (42.80, 192.93)	0.16
PM_2.5_ aluminum, μg/m^3^	5.42 (1.78, 16.51)	7.58 (2.49, 23.08)	4.61 (1.51, 14.04)	2.54 (0.83, 7.72)	7.01 (2.30, 21.34)	7.19 (2.36, 21.89)	1.25 (0.41, 3.81)	0.79 (0.26, 2.40)	0.81
PM_2.5_ black carbon, μg/m^3^	6.84 (2.73, 17.16)	9.14 (3.64, 22.92)	34.02 (13.56, 85.36)	14.34 (5.72, 35.99)	34.09 (13.59, 85.53)	28.99 (11.55, 72.73)	9.82 (3.92, 24.65)	9.55 (3.81, 23.97)	0.62
PM_2.5_ barium, μg/m^3^	0.25 (0.096, 0.65) ^D^	0.38 (0.15, 0.95) ^D^	0.29 (0.11, 0.73) ^D^	0.20 (0.079, 0.50) ^D^	0.45 (0.18, 1.15) ^D^	0.34 (0.13, 0.87) ^D^	0.046 (0.013, 0.16) ^D^	0.061 (0.018, 0.20) ^D^	0.81 ^E^
PM_2.5_ brown carbon, μg/m^3^	4.53 (0.31, 67.26) ^D^	2.32 (0.14, 37.57) ^D^	0.17 (0.0079, 3.54) ^D^	1.54 (0.095, 25.13) ^D^	1.12 (0.061, 20.88) ^D^	0.36 (0.015, 8.99) ^D^	0.61 (0.036, 10.10) ^D^	0.17 (0.0082, 3.65) ^D^	0.61 ^E^
PM_2.5_ bromine, μg/m^3^	1.00 (Reference)	^F^	6.89 (0.80, ∞) ^G,H^	^F^	1.00 (0.05, ∞) ^G,H^	^F^	^F^	^F^	^I^
PM_2.5_ caesium, μg/m^3^	0.13 (0.049, 0.33) ^D^	0.18 (0.074, 0.44) ^D^	0.13 (0.055, 0.33) ^D^	0.050 (0.016, 0.16) ^D^	0.15 (0.060, 0.35) ^D^	0.21 (0.086, 0.50) ^D^	0.046 (0.014, 0.15) ^D^	0.051 (0.016, 0.16) ^D^	0.50 ^E^
PM_2.5_ calcium, μg/m^3^	1.66 (0.65, 4.21)	2.03 (0.80, 5.14)	1.57 (0.62, 3.97)	0.82 (0.32, 2.07)	1.63 (0.64, 4.13)	1.46 (0.58, 3.71)	0.47 (0.19, 1.20)	0.27 (0.11, 0.68)	0.76
PM_2.5_ chlorine, μg/m^3^	0.17 (0.055, 0.54) ^D^	0.12 (0.034, 0.41) ^D^	0.79 (0.25, 2.49) ^D^	0.14 (0.044, 0.43) ^D^	0.62 (0.20, 1.95) ^D^	0.17 (0.053, 0.56) ^D^	0.17 (0.053, 0.52) ^D^	0.14 (0.043, 0.46) ^D^	0.50 ^E^
PM_2.5_ chromium, μg/m^3^	0.064 (0.034, 0.12) ^D^	0.10 (0.062, 0.16) ^D^	0.0044 ^D,J^	0.067 (0.037, 0.12) ^D^	0.088 (0.055, 0.14) ^D^	0.13 (0.085, 0.21) ^D^	0.0045 ^D,J^	0.069 (0.038, 0.12) ^D^	>0.99 ^E^
PM_2.5_ cobalt, μg/m^3^	0.016 (0.0040, 0.064) ^D^	0.013 (0.0032, 0.053) ^D^	0.020 (0.0057, 0.069) ^D^	0.015 (0.0040, 0.054) ^D^	0.022 (0.0059, 0.078) ^D^	0.015 (0.0035, 0.067) ^D^	0.0000023 ^D,J^	0.0053 (0.0010, 0.028) ^D^	> 0.99 ^E^
PM_2.5_ iron, μg/m^3^	3.95 (1.31, 11.88)	5.87 (1.95, 17.64)	4.71 (1.57, 14.15)	2.46 (0.82, 7.39)	6.52 (2.17, 19.60)	6.69 (2.22, 20.11)	1.15 (0.38, 3.46)	0.64 (0.21, 1.92)	0.72
PM_2.5_ lead, μg/m^3^	0.00030 ^D,J^	0.016 (0.0069, 0.038) ^D^	0.035 (0.018, 0.068) ^D^	0.013 (0.0056, 0.032) ^D^	0.022 (0.011, 0.044) ^D^	0.075 (0.040, 0.14) ^D^	0.00031 ^D,J^	0.014 (0.0059, 0.033) ^D^	0.03 ^E^
PM_2.5_ magnesium, μg/m^3^	0.16 (0.10, 0.24) ^D^	0.12 (0.072, 0.20) ^D^	0.12 (0.080, 0.19) ^D^	0.12 (0.074, 0.18) ^D^	0.0073 ^D,J^	0.0074 ^D,J^	0.13 (0.081, 0.20) ^D^	0.090 (0.052, 0.16) ^D^	0.95 ^E^
PM_2.5_ manganese, μg/m^3^	0.044 (0.017, 0.12) ^D^	0.070 (0.027, 0.18) ^D^	0.058 (0.023, 0.15) ^D^	0.016 (0.0055, 0.046) ^D^	0.076 (0.030, 0.20) ^D^	0.069 (0.027, 0.18) ^D^	0.010 (0.0029, 0.034) ^D^	0.010 (0.0031, 0.035) ^D^	0.35 ^E^
PM_2.5_ nickel, μg/m^3^	1.00 (Reference)	1.00 (0.03, 30.58) ^G^	0.33 (0.00, 3.22) ^G,H^	0.38 (< 0.01, 12.69) ^G^	1.00 (0.03, 30.58) ^G^	0.38 (< 0.01, 12.69) ^G^	0.33 (0.00, 3.22) ^G,H^	0.38 (< 0.01, 12.69) ^G^	1.00 ^K^
PM_2.5_ potassium, μg/m^3^	1.75 (0.73, 4.18)	2.50 (1.04, 5.98)	3.37 (1.41, 8.06)	1.55 (0.65, 3.70)	4.39 (1.83, 10.49)	4.93 (2.06, 11.79)	0.73 (0.31, 1.75)	0.47 (0.20, 1.12)	0.53
PM_2.5_ rubidium, μg/m^3^	0.0087 (0.0021, 0.036) ^D^	0.017 (0.0054, 0.057) ^D^	0.023 (0.0079, 0.068) ^D^	0.000011 ^D,J^	0.026 (0.0089, 0.077) ^D^	0.028 (0.0090, 0.086) ^D^	0.000011 ^D,J^	0.000011 ^D,J^	0.96 ^E^
PM_2.5_ silicon, μg/m^3^	9.87 (3.61, 27.00)	15.13 (5.53, 41.39)	11.34 (4.15, 31.04)	6.75 (2.47, 18.47)	14.97 (5.47, 40.96)	16.01 (5.85, 43.82)	3.43 (1.25, 9.39)	1.78 (0.65, 4.87)	0.66
PM_2.5_ sodium, μg/m^3^	1.00 (Reference)	^F^	6.89 (0.80, ∞) ^G,H^	1.00 (0.05, ∞) ^G,H^	^F^	1.00 (0.05, ∞) ^G,H^	^F^	^F^	0.31 ^K^
PM_2.5_ strontium, μg/m^3^	1.00 (Reference)	0.38 (< 0.01, 12.69) ^G^	0.38 (< 0.01, 12.69)^G^	0.33 (0.00, 3.22) ^G,H^	2.60 (0.08, 235.00) ^G^	0.38 (< 0.01, 12.69) ^G^	0.33 (0.00, 3.22) ^G,H^	0.33 (0.00, 3.22) ^G,H^	1.00 ^K^
PM_2.5_ sulfur, μg/m^3^	1.53 (1.02, 2.31)	1.57 (1.05, 2.37)	2.66 (1.77, 4.01)	2.20 (1.46, 3.31)	3.70 (2.46, 5.56)	4.99 (3.32, 7.50)	1.41 (0.93, 2.11)	1.48 (0.98, 2.22)	0.68
PM_2.5_ titanium, μg/m^3^	0.48 (0.16, 1.44) ^D^	0.65 (0.22, 1.95) ^D^	0.53 (0.18, 1.59) ^D^	0.26 (0.088, 0.78) ^D^	0.82 (0.27, 2.43) ^D^	0.90 (0.30, 2.67) ^D^	0.088 (0.028, 0.27) ^D^	0.072 (0.022, 0.24) ^D^	0.82 ^E^
PM_2.5_ vanadium, μg/m^3^	1.00 (Reference)	0.33 (0.00, 3.22) ^G,H^	0.33 (0.00, 3.22) ^G,H^	0.33 (0.00, 3.22) ^G,H^	1.00 (0.03, 30.58) ^G^	0.38 (< 0.01, 12.69) ^G^	0.33 (0.00, 3.22) ^G,H^	0.33 (0.00, 3.22) ^G,H^	1.00 ^K^
PM_2.5_ zinc, μg/m^3^	0.040 (0.019, 0.085) ^D^	0.049 (0.023, 0.10) ^D^	0.11 (0.052, 0.23) ^D^	0.041 (0.020, 0.087) ^D^	0.10 (0.049, 0.22) ^D^	0.12 (0.058, 0.26) ^D^	0.011 (0.0047, 0.026) ^D^	0.015 (0.0060, 0.036) ^D^	0.31 ^E^
Relative humidity, %	27.04 (23.56, 31.05)	18.82 (16.39, 21.60)	37.20 (32.41, 42.71)	33.61 (29.28, 38.58)	52.05 (45.34, 59.75)	55.96 (47.71, 65.62)	65.20 (56.79, 74.85)	65.06 (56.67, 74.68)	0.02
Temperature, °C	29.08 (27.11, 31.19)	34.15 (31.84, 36.63)	25.76 (24.02, 27.63)	27.27 (25.43, 29.25)	27.92 (26.03, 29.94)	25.89 (23.88, 28.07)	22.71 (21.18, 24.36)	21.51 (20.06, 23.07)	0.01

Abbreviations: CI, confidence interval; GM, geometric mean; PM_2.5_, particulate matter with an aerodynamic diameter less than 2.5 μm; ^A^ Eleven air pollutants measured above detection limits in less than 10% of samples. The names, detection limits, and summary statistics for these air pollutants are included in Supplemental Table S1; ^B^ Estimated via linear regression models of the natural logarithm transformed values; ^C^ Interactions were tested via Type III. F tests; ^D^ Estimated via Tobit regression models of the natural logarithm transformed values; ^E^ Interactions were tested via Type III. χ^2^ tests; ^F^ Unable to estimate odds ratio and 95% CI; ^G^ Odds ratio and 95% CI; estimated via exact unconditional logistic regression models; ^H^ Median unbiased estimate; ^I^ Unable to estimate p-value; ^J^ Unable to estimate 95% CI; ^K^ Interactions were tested via exact score tests.

## Supplementary Materials

The following are available online at https://www.mdpi.com/1660-4601/16/21/4114/s1, Table S1: Detection limits and summary statistics for air pollutants, temperature, and RH measured at on-site housing at brick kilns in Bhaktapur, Nepal, May 2018; Table S2: Associations between characteristics and PM_2.5_, PM_2.5_ aluminum, and PM2.5 black carbon air concentrations measured at on-site homes at brick kilns in Bhaktapur, Nepal, May 2018; Table S3: Associations between characteristics and PM_2.5_ barium, PM_2.5_ brown carbon, and PM_2.5_ bromine air concentrations measured at on-site homes at brick kilns in Bhaktapur, Nepal, May 2018; Table S4: Associations between characteristics and PM_2.5_ caesium, PM_2.5_ calcium, and PM_2.5_ chlorine air concentrations measured at on-site homes at brick kilns in Bhaktapur, Nepal, May 2018; Table S5: Associations between characteristics and PM_2.5_ chromium, PM_2.5_ cobalt, and PM_2.5_ iron air concentrations measured at on-site homes at brick kilns in Bhaktapur, Nepal, May 2018; Table S6: Associations between characteristics and PM_2.5_ lead, PM_2.5_ magnesium, and PM_2.5_ manganese air concentrations measured at on-site homes at brick kilns in Bhaktapur, Nepal, May 2018; Table S7: Associations between characteristics and PM_2.5_ nickel, PM_2.5_ potassium, and PM_2.5_ rubidium air concentrations measured at on-site homes at brick kilns in Bhaktapur, Nepal, May 2018; Table S8: Associations between characteristics and PM_2.5_ silicon, PM_2.5_ sodium, and PM_2.5_ strontium air concentrations measured at on-site homes at brick kilns in Bhaktapur, Nepal, May 2018; Table S9: Associations between characteristics and PM_2.5_ sulfur, PM_2.5_ titanium, and PM_2.5_ vanadium air concentrations measured at on-site homes at brick kilns in Bhaktapur, Nepal, May 2018; Table S10: Associations between characteristics and PM_2.5_ zinc air concentrations, relative humidity, and temperature measured at on-site homes at brick kilns in Bhaktapur, Nepal, May 2018.

## References

[1] Joshi S.K., Dahal P., Poudel A., Sherpa H. (2013). Work related injuries and musculoskeletal disorders among child workers in the brick kilns of Nepal. Int. J. Occup. Saf. Health.

[2] Sanjel S., Thygerson S.M., Khanal S.N., Joshi S.K. (2016). Environmental and Occupational Pollutants and Their Effects on Health among Brick Kiln Workers. Open J. Saf. Sci. Technol..

[3] Larmar S., O’Leary P., Chui C., Benfer K., Zug S., Jordan L.P. (2017). Hazardous child labor in Nepal: The case of brick kilns. Child Abus. Negl..

[4] Das S., Hasan S.Q., Akhter R., Huque S., Khandaker S., Gorapi Z.H., Shahriar M. (2017). Socioeconomic conditions and health hazards of brick field workers: A case study of Mymensingh brick industrial area of Bangladesh. J. Public Health Epidemiol..

[5] Kainth G.S. (2009). Push and pull factors of migration: A case of brick kiln industry of Punjab State. Asia-Pac. J. Soc. Sci..

[6] Dharmalingam A. (1995). Conditions of brick workers in south Indian village. Econ. Political Wkly..

[7] Haack B.N., Khatiwada G. (2007). Rice and bricks: Environmental issues and mapping of the unusual crop rotation pattern in the Kathmandu Valley, Nepal. Environ. Manag..

[8] ENPHO (2001). A Study on Status of Brick Industry in the Kathmandu Valley.

[9] Raut A.K. (2003). Brick kilns in Kathmandu valley: Current status, environmental impacts and future options. Himalyan J. Sci..

[10] Sanjel S., Khanal S.N., Thygerson S.M., Carter W.S., Johnston J.D., Joshi S.K. (2017). Respiratory symptoms and illnesses related to the concentration of airborne particulate matter among brick kiln workers in Kathmandu valley, Nepal. Ann. Occup. Environ. Med..

[11] Sanjel S., Khanal S.N., Thygerson S.M., Carter W., Johnston J.D., Joshi S.K. (2017). Exposure to respirable silica among clay brick workers in Kathmandu valley, Nepal. Arch. Environ. Occup. Health.

[12] Bruce N., Perez-Padilla R., Albalak R. (2000). Indoor air pollution in developing countries: A major environmental and public health challenge. Bull. World Health Organ..

[13] Shakya K.M., Rupakheti M., Shahi A., Maskey R., Pradhan B., Panday A., Puppala S.P., Lawrence M., Peltier R.E. (2017). Near-road sampling of PM2.5, BC, and fine-particle chemical components in Kathmandu Valley, Nepal. Atmos. Chem. Phys..

[14] Saud B., Paudel G. (2018). The Threat of Ambient Air Pollution in Kathmandu, Nepal. J. Environ. Public Health.

[15] Nepal S., Mahapatra P.S., Adhikari S., Shrestha S., Sharma P., Shrestha K.L., Pradhan B.B., Puppala S.P. (2019). A Comparative Study of Stack Emissions from Straight-Line and Zigzag Brick Kilns in Nepal. Atmosphere.

[16] Gautam D., Rodrigues H., Bhetwal K.K., Neupane P., Sanada Y. (2016). Common structural and construction deficiencies of Nepalese buildings. Innov. Infrastruct. Solut..

[17] Angster S., Fielding E.J., Wesnousky S., Pierce I., Chamlagain D., Gautam D., Upreti B.N., Kumahara Y., Nakata T. (2015). Field Reconnaissance after the 25 April 2015 M 7.8 Gorkha Earthquake. Seismol. Res. Lett..

[18] Wallace L.A., Mitchell H., O’Connor G.T., Neas L., Lippmann M., Kattan M., Koenig J., Stout J.W., Vaughn B.J., Wallace D. (2003). Particle concentrations in inner-city homes of children with asthma: The effect of smoking, cooking, and outdoor pollution. Environ. Health Perspect..

[19] Dockery D.W., Spengler J.D. (1981). Indoor-outdoor relationships of respirable sulfates and particles. Atmos. Environ. (1967).

[20] Kingham S., Briggs D., Elliott P., Fischer P., Lebret E. (2000). Spatial variations in the concentrations of traffic-related pollutants in indoor and outdoor air in Huddersfield, England. Atmos. Environ..

[21] Pope C.A., Dockery D.W. (2006). Health effects of fine particulate air pollution: Lines that connect. J. Air Waste Manag. Assoc..

[22] Woodruff T.J., Parker J.D., Schoendorf K.C. (2006). Fine particulate matter (PM2. 5) air pollution and selected causes of postneonatal infant mortality in California. Environ. Health Perspect..

[23] Loomis D., Castillejos M., Gold D.R., McDonnell W., Borja-Aburto V.H. (1999). Air pollution and infant mortality in Mexico City. Epidemiology.

[24] Karr C., Lumley T., Schreuder A., Davis R., Larson T., Ritz B., Kaufman J. (2006). Effects of subchronic and chronic exposure to ambient air pollutants on infant bronchiolitis. Am. J. Epidemiol..

[25] Ostro B., Broadwin R., Green S., Feng W.-Y., Lipsett M. (2005). Fine particulate air pollution and mortality in nine California counties: Results from CALFINE. Environ. Health Perspect..

[26] Laden F., Schwartz J., Speizer F.E., Dockery D.W. (2006). Reduction in fine particulate air pollution and mortality: Extended follow-up of the Harvard Six Cities study. Am. J. Respir. Crit. Care Med..

[27] WHO (2014). WHO Indoor Air Quality Guidelines: Household Fuel Combustion.

[28] Rehfuess E., Corvalan C., Neira M. (2006). Indoor air pollution: 4000 deaths a day must no longer be ignored. Bull. World Health Organ..

[29] Bell M.L., Belanger K., Ebisu K., Gent J.F., Lee H.J., Koutrakis P., Leaderer B.P. (2010). Prenatal exposure to fine particulate matter and birth weight: Variations by particulate constituents and sources. Epidemiol. (Camb. Mass.).

[30] Ebisu K., Bell M.L. (2012). Airborne PM2. 5 chemical components and low birth weight in the northeastern and mid-Atlantic regions of the United States. Environ. Health Perspect..

[31] Ostro B., Roth L., Malig B., Marty M. (2008). The effects of fine particle components on respiratory hospital admissions in children. Environ. Health Perspect..

[32] Code of Federal Regulations Title 45, Part 46. Protection of Human Subjects.

[33] Lawless P.A., Rodes C.E., Ensor D.S. (2004). Multiwavelength absorbance of filter deposits for determination of environmental tobacco smoke and black carbon. Atmos. Environ..

[34] Sloan C.D., Weber F.X., Bradshaw R.K., Philipp T.J., Barber W.B., Palmer V.L., Graul R.J., Tuttle S.C., Chartier R.T., Johnston J.D. (2017). Elemental analysis of infant airborne particulate exposures. J. Expo. Sci. Environ. Epidemiol..

[35] United States Environmental Protection Agency (1999). Compendium Method IO-3.3: Determination of Metals in Ambient Particulate Matter Using X-ray Fluorescence (XRF) Sprectroscopy.

[36] Beard J.D., Erdely A., Dahm M.M., de Perio M.A., Birch M.E., Evans D.E., Fernback J.E., Eye T., Kodali V., Mercer R.R. (2018). Carbon nanotube and nanofiber exposure and sputum and blood biomarkers of early effect among US workers. Environ. Int..

[37] Lubin J.H., Colt J.S., Camann D., Davis S., Cerhan J.R., Severson R.K., Bernstein L., Hartge P. (2004). Epidemiologic evaluation of measurement data in the presence of detection limits. Environ. Health Perspect..

[38] Maithel S., Kumar S., Lalchandani D. (2014). Factsheets about Brick Kilns in South and South-East Asia.

[39] World Health Organization (2006). Air Quality Guidelines: Global Update 2005: Particulate Matter, Ozone, Nitrogen Dioxide, and Sulfur Dioxide.

[40] GMAO MERRA-2 Modern-Era Retrospective Analysis. https://gmao.gsfc.nasa.gov/reanalysis/MERRA-2/.

[41] Li C., Kang S., Chen P., Zhang Q., Guo J., Mi J., Basang P., Luosang Q., Smith K.R. (2012). Personal PM2.5 and indoor CO in nomadic tents using open and chimney biomass stoves on the Tibetan Plateau. Atmos. Environ..

[42] Ostro B., Feng W.-Y., Broadwin R., Green S., Lipsett M. (2006). The effects of components of fine particulate air pollution on mortality in California: Results from CALFINE. Environ. Health Perspect..

[43] Cao J.-J., Shen Z.-X., Chow J.C., Watson J.G., Lee S.-C., Tie X.-X., Ho K.F., Wang G.H., Han Y.M. (2012). Winter and summer PM2. 5 chemical compositions in fourteen Chinese cities. J. Air Waste Manag. Assoc..

[44] Chen X.-C., Jahn H.J., Engling G., Ward T.J., Kraemer A., Ho K.-F., Yim S.H.L., Chan C.Y. (2017). Chemical characterization and sources of personal exposure to fine particulate matter (PM2. 5) in the megacity of Guangzhou, China. Environ. Pollut..

[45] Chen X., Zhang Z., Engling G., Zhang R., Tao J., Lin M., Sang X., Chan C., Li S., Li Y. (2014). Characterization of fine particulate black carbon in Guangzhou, a megacity of South China. Atmos. Pollut. Res..

[46] Zhou M., Qiao L., Zhu S., Li L., Lou S., Wang H., Wang Q., Tao S., Huang C., Chen C. (2016). Chemical characteristics of fine particles and their impact on visibility impairment in Shanghai based on a 1-year period observation. J. Environ. Sci..

