# Supplementary material for: Air-Quality Assessment of On-Site Brick-Kiln Worker Housing in Bhaktapur, Nepal: Chemical Speciation of Indoor and Outdoor PM2.5 Pollution
Source: Int J Environ Res Public Health. 2019 Oct 25;16(21):4114. doi: 10.3390/ijerph16214114 (PMC6862110; doi:10.3390/ijerph16214114)
Supplement: Supplementary file 1 [file ijerph-16-04114-s001.pdf]

Supplementary

Table S1. Detection limits and summary statistics for air pollutants, temperature, and RH measured at on-site housing at brick kilns in Bhaktapur, Nepal, May 2018.

Air Pollutant or Weather Variable	Total Samples								
	Missing, n	DL Mass (µg)	DL Concentration Range	Below DL, n (%)	Above DL				
					n (%)	GM [A]	95% CI [A]	Min [B]	Max [B]
$PM_{2.5}$, µg/m^3		12.00[C]	13.90, 19.60[C]	[D]	[D]	[D]	[D]	[D]	[D]
$PM_{2.5}$ aluminum, µg/m^3		0.089	0.10, 0.15	[D]	[D]	[D]	[D]	[D]	[D]
$PM_{2.5}$ antimony, µg/m^3		0.26	0.30, 0.42	31 (97)	1 (3)	0.067[E]	0.0027, 1.58[E]	0.50	0.50
$PM_{2.5}$ arsenic, µg/m^3		0.0062	0.0072, 0.010	29 (91)	3 (9)	0.00039[E]	0.000011, 0.014[E]	0.010	0.084
$PM_{2.5}$ black carbon, µg/m^3		0.04	0.046, 0.065	[D]	[D]	[D]	[D]	[D]	[D]
$PM_{2.5}$ barium, µg/m^3		0.073	0.084, 0.12	[D]	[D]	[D]	[D]	[D]	[D]
$PM_{2.5}$ brown carbon, µg/m^3		0.20	0.23, 0.33	[D]	[D]	[D]	[D]	[D]	[D]
$PM_{2.5}$ bromine, µg/m^3		0.017[C]	0.019, 0.027[C]	[D]	[D]	[D]	[D]	[D]	[D]
$PM_{2.5}$ cadmium, µg/m^3		0.11	0.13, 0.19	32 (100)	0 (0)	NA	NA	NA	NA
$PM_{2.5}$ caesium, µg/m^3		0.076	0.088, 0.12	[D]	[D]	[D]	[D]	[D]	[D]
$PM_{2.5}$ calcium, µg/m^3		0.050	0.058, 0.082	[D]	[D]	[D]	[D]	[D]	[D]
$PM_{2.5}$ cerium, µg/m^3		0.065	0.075, 0.11	31 (97)	1 (3)	0.000020[E]	0.0000000000016, 252.42[E]	0.61	0.61
$PM_{2.5}$ chlorine, µg/m^3		0.052	0.060, 0.085	[D]	[D]	[D]	[D]	[D]	[D]
$PM_{2.5}$ chromium, µg/m^3		0.071[C]	0.082, 0.12[C]	[D]	[D]	[D]	[D]	[D]	[D]
$PM_{2.5}$ cobalt, µg/m^3		0.0090	0.010, 0.015	[D]	[D]	[D]	[D]	[D]	[D]
$PM_{2.5}$ copper, µg/m^3		0.011	0.012, 0.018	30 (94)	2 (6)	0.00025[E]	0.00000084, 0.077[E]	0.032	0.082
$PM_{2.5}$ indium, µg/m^3		0.11	0.12, 0.17	32 (100)	0 (0)	NA	NA	NA	NA
$PM_{2.5}$ iron, µg/m^3		0.020[C]	0.023, 0.033[C]	[D]	[D]	[D]	[D]	[D]	[D]
$PM_{2.5}$ lead, µg/m^3		0.017	0.020, 0.029	[D]	[D]	[D]	[D]	[D]	[D]
$PM_{2.5}$ magnesium, µg/m^3		0.095[C]	0.11, 0.16[C]	[D]	[D]	[D]	[D]	[D]	[D]
$PM_{2.5}$ manganese, µg/m^3		0.013	0.015, 0.021	[D]	[D]	[D]	[D]	[D]	[D]
$PM_{2.5}$ nickel, µg/m^3		0.0080	0.0092, 0.013	[D]	[D]	[D]	[D]	[D]	[D]
$PM_{2.5}$ phosphorus, µg/m^3		0.11	0.12, 0.17	31 (97)	1 (3)	0.082[E]	0.032, 0.21[E]	0.16	0.16
$PM_{2.5}$ potassium, µg/m^3		0.048	0.056, 0.079	[D]	[D]	[D]	[D]	[D]	[D]
$PM_{2.5}$ rubidium, µg/m^3		0.013	0.015, 0.021	[D]	[D]	[D]	[D]	[D]	[D]
$PM_{2.5}$ selenium, µg/m^3		0.0086	0.010, 0.014	32 (100)	0 (0)	NA	NA	NA	NA
$PM_{2.5}$ silicon, µg/m^3		0.064	0.074, 0.10	[D]	[D]	[D]	[D]	[D]	[D]

Air Pollutant or Weather Variable	Total Samples								
	Missing, n	DL Mass (μg)	DL Concentration Range	Below DL, n (%)	Above DL				
					n (%)	GM [A]	95% CI [A]	Min [B]	Max [B]
$PM_{2.5}$ silver, μg/m³		0.087	0.10, 0.14	32 (100)	0 (0)	NA	NA	NA	NA
$PM_{2.5}$ sodium, μg/m³		0.21	0.24, 0.34	D	D	D	D	D	D
$PM_{2.5}$ strontium, μg/m³		0.016	0.018, 0.026	D	D	D	D	D	D
$PM_{2.5}$ sulfur, μg/m³		0.083[C]	0.097, 0.14[C]	D	D	D	D	D	D
$PM_{2.5}$ tin, μg/m³		0.14	0.16, 0.22	30 (94)	2 (6)	0.067[E]	0.017, 0.26[E]	0.21	0.28
$PM_{2.5}$ titanium, μg/m³		0.035	0.041, 0.058	D	D	D	D	D	D
$PM_{2.5}$ vanadium, μg/m³		0.026	0.030, 0.042	D	D	D	D	D	D
$PM_{2.5}$ zinc, μg/m³		0.012	0.014, 0.019	D	D	D	D	D	D
$PM_{2.5}$ zirconium, μg/m³		0.022	0.026, 0.036	31 (97)	1 (3)	0.00000017[E]	0.0000000000000000093, 3011.77[E]	0.50	0.50
Relative humidity, %	1	NA	NA	D	D	D	D	D	D
Temperature, °C	1	NA	NA	D	D	D	D	D	D

Abbreviations: CI, confidence interval; DL, detection limit; GM, geometric mean; Max, maximum; Min, minimum; NA, not applicable; PM2.5, particulate matter with an aerodynamic diameter less than 2.5 μm; A Estimated via linear regression models of the natural logarithm transformed values; B Calculated from samples that had values above detection limits; C Maximum mass and concentration ranges from blank samples that measured above detection limits were used instead of detection limit masses and concentration ranges; D Summary statistics are included in Table 2; E Estimated via Tobit regression models of the natural logarithm transformed values.

Table S2. Associations between characteristics and $PM_{2.5}$, $PM_{2.5}$ aluminum, and $PM_{2.5}$ black carbon air concentrations measured at on-site homes at brick kilns in Bhaktapur, Nepal, May 2018.

Characteristic	Air Pollutant								
	$PM_{2.5}$, μg/m³			$PM_{2.5}$ aluminum, μg/m³			$PM_{2.5}$ black carbon, μg/m³		
	GM [A]	95% CI [A]	*p*-value [A]	GM [A]	95% CI [A]	*p*-value [A]	GM [A]	95% CI [A]	*p*-value [A]
Kiln number									
1	165.22	95.94, 284.51		6.41	3.06, 13.45		7.91	4.24, 14.75	
2	211.93	123.07, 364.96		3.42	1.63, 7.17		22.09	11.84, 41.23	
3	364.61	211.74, 627.87		7.10	3.38, 14.89		31.44	16.84, 58.67	
4	91.06	52.88, 156.81	0.009[B]	0.99	0.47, 2.08	0.002[C]	9.69	5.19, 18.09	0.009[D]
Type of home									
Worker	146.44	94.57, 226.74		2.48	1.33, 4.63		14.80	8.79, 24.89	
Fire master	232.84	150.37, 360.54	0.14	5.02	2.69, 9.38	0.11	15.59	9.26, 26.22	0.89
Location of sample									
Indoor	182.80	116.10, 287.84		3.85	2.01, 7.37		16.71	9.95, 28.05	
Outdoor	186.52	118.46, 293.69	0.95	3.23	1.69, 6.19	0.70	13.80	8.22, 23.17	0.60
Size of house, 50 feet²	1.12[E]	0.69, 1.83[E]	0.63	1.20[E]	0.59, 2.46[E]	0.60	1.27[E]	0.72, 2.24[E]	0.40
Size of house, m²	1.02[E]	0.92, 1.14[E]	0.64	1.04[E]	0.89, 1.21[E]	0.61	1.05[E]	0.93, 1.19[E]	0.40
How long lived in house, two months	1.06[E]	0.98, 1.15[E]	0.16	1.14[E]	1.01, 1.28[E]	0.03	0.96[E]	0.87, 1.07[E]	0.46
How many people live in house	0.91[E]	0.76, 1.09[E]	0.29	1.03[E]	0.79, 1.34[E]	0.83	0.81[E]	0.66, 0.99[E]	0.04
Occupant density, 10 residents/100 m²	0.94[E]	0.87, 1.01[E]	0.09	0.95[E]	0.84, 1.07[E]	0.38	0.90[E]	0.83, 0.99[E]	0.03
How many children 0-18 years-old live in house									
0	243.23	162.65, 363.72		4.27	2.31, 7.88		18.31	11.27, 29.77	
1–3	144.09	83.98, 247.23	0.12	3.79	1.66, 8.63	0.81	10.38	5.41, 19.92	0.16
How many children under 6 years-old live in house									
0	243.23	162.94, 363.08		4.27	2.32, 7.84		18.31	11.11, 30.18	

Characteristic	Air Pollutant								
	$PM_{2.5}$, μg/m³			$PM_{2.5}$ aluminum, μg/m³			$PM_{2.5}$ black carbon, μg/m³		
	GM [A]	95% CI [A]	*p*-value [A]	GM [A]	95% CI [A]	*p*-value [A]	GM [A]	95% CI [A]	*p*-value [A]
1–3	118.59	65.02, 216.29	0.05	2.74	1.10, 6.82	0.41	11.98	5.66, 25.34	0.34
Primary fuel used for cooking									
Gas only	101.83	51.22, 202.45		1.26	0.46, 3.50		10.18	4.49, 23.07	
Wood only	243.99	164.08, 362.81		5.18	1.87, 14.31		18.66	11.64, 29.93	
Other [F]	161.26	81.11, 320.60	0.09	4.56	2.54, 8.21	0.08	10.18	4.49, 23.08	0.27
Type of heating source in the home									
Electricity	144.40	96.60, 215.87		2.02	1.16, 3.49		14.19	8.33, 24.16	
Other [G] or none	225.06	127.46, 397.40	0.20	5.32	2.45, 11.57	0.05	15.46	7.28, 32.84	0.85
Type of non-electric light source in the home									
Candle	197.20	118.72, 327.55		3.22	1.56, 6.67		16.60	9.93, 27.76	
Generator	165.22	84.43, 323.29		6.41	2.45, 16.79		7.91	4.00, 15.61	
Other[H] or none	194.03	89.38, 421.23	0.91	2.23	0.73, 6.79	0.32	19.09	8.71, 41.87	0.15
Any smokers living in the home									
No	283.51	165.59, 485.42		10.03	4.98, 20.17		11.37	5.99, 21.58	
Yes	153.80	105.15, 224.96	0.07	2.17	1.33, 3.56	0.001	16.63	10.57, 26.16	0.33
How many smokers living in the home									
0	283.51	166.22, 483.57		10.03	4.62, 21.76		11.37	6.31, 20.46	
1–2	116.20	63.97, 211.10		2.32	0.97, 5.51		11.45	5.93, 22.09	
3–4	199.37	100.07, 397.22	0.09	2.79	1.03, 7.58	0.03[I]	20.28	9.49, 43.32	0.41
How many smokers living in the home regularly smoke inside the home									

Characteristic	Air Pollutant								
	$PM_{2.5}$, μg/m³			$PM_{2.5}$ aluminum, μg/m³			$PM_{2.5}$ black carbon, μg/m³		
	GM [A]	95% CI [A]	*p*-value [A]	GM [A]	95% CI [A]	*p*-value [A]	GM [A]	95% CI [A]	*p*-value [A]
0–1	191.82	114.36, 321.77		2.03	1.00, 4.12		24.50	13.20, 45.47	
2–4	103.64	49.87, 215.38	0.17	2.39	0.88, 6.53	0.78	8.81	3.67, 21.12	0.06

Abbreviations: CI, confidence interval; GM, geometric mean; $PM_{2.5}$, particulate matter with an aerodynamic diameter less than 2.5 μm; [A] Estimated via linear regression models of the natural logarithm transformed values; [B] Using the Tukey method to adjust for multiple comparisons, p-values for tests of pairwise differences among kilns were as follows: 1 vs. 2: 0.91, 1 vs. 3: 0.17, 1 vs. 4: 0.40, 2 vs. 3: 0.48, 2 vs. 4: 0.13, and 3 vs. 4: 0.005; [C] Using the Tukey method to adjust for multiple comparisons, p-values for tests of pairwise differences among kilns were as follows: 1 vs. 2: 0.61, 1 vs. 3: >0.99, 1 vs. 4: 0.006, 2 vs. 3: 0.49, 2 vs. 4: 0.10, and 3 vs. 4: 0.003; [D] Using the Tukey method to adjust for multiple comparisons, *p*-values for tests of pairwise differences among kilns were as follows: 1 vs. 2: 0.10, 1 vs. 3: 0.02, 1 vs. 4: 0.96, 2 vs. 3: 0.85, 2 vs. 4: 0.25, and 3 vs. 4: 0.05; [E] Exponentiated regression coefficient and 95% CI (i.e., GM air pollutant concentration ratio for a specified change in the independent variable or exp(β) – 1 = percent change in GM air pollutant concentration for a specified change in the independent variable); [F] Includes coal and wood, gas and wood; [G] Includes lightbulb, line cable; [H] Includes candle and torch, fuel; [I] Using the Tukey method to adjust for multiple comparisons, p-values for tests of pairwise differences among categories of how many smokers living in the home were as follows: 0 vs. 1–2: 0.04, 0 vs. 3–4: 0.11, and 1–2 vs. 3–4: 0.95.

Table S3. Associations between characteristics and $PM_{2.5}$ barium, $PM_{2.5}$ brown carbon, and $PM_{2.5}$ bromine air concentrations measured at on-site homes at brick kilns in Bhaktapur, Nepal, May 2018.

Characteristic	Air Pollutant								
	$PM_{2.5}$ barium, μg/m³			$PM_{2.5}$ brown carbon, μg/m³			$PM_{2.5}$ bromine, μg/m³		
	GM [A]	95% CI [A]	*p*-value [A]	GM [A]	95% CI [A]	*p*-value [A]	Exact OR [B]	Exact 95% CI [B]	Exact *p*-value [B]
Kiln number									
1	0.31	0.16, 0.60		3.25	0.44, 23.81		1.00	Reference	
2	0.24	0.12, 0.47		0.53	0.064, 4.38		4.86 [C]	0.65, ∞	
3	0.39	0.20, 0.77		0.63	0.066, 6.00		1.00 [C]	0.05, ∞	
4	0.052	0.021, 0.13	0.003 [D]	0.33	0.039, 2.75	0.43	[E]	[E]	0.16
Type of home									
Worker	0.14	0.078, 0.25		0.51	0.11, 2.44		1.00	Reference	
Fire master	0.30	0.18, 0.53	0.06	1.18	0.25, 5.51	0.45	1.00	0.06, 15.62	1.00
Location of sample									
Indoor	0.21	0.12, 0.38		0.86	0.18, 4.05		1.00	Reference	
Outdoor	0.20	0.11, 0.37	0.94	0.68	0.13, 3.41	0.83	0.16 [C]	0.00, 1.01	0.10
Size of house, 50 feet²	1.06 [F]	0.55, 2.04 [F]	0.86	0.89 [F]	0.16, 5.08 [F]	0.89	1.91	0.43, 9.97	0.41
Size of house, m²	1.01 [F]	0.88, 1.17 [F]	0.88	0.97 [F]	0.67, 1.42 [F]	0.89	1.15	0.83, 1.65	0.41
How long lived in house, two months	1.14 [F]	1.04, 1.26 [F]	0.007	1.09 [F]	0.83, 1.44 [F]	0.54	0.68	0.05, 1.24	0.65
How many people live in house	0.98 [F]	0.77, 1.25 [F]	0.87	1.64 [F]	0.92, 2.93 [F]	0.09	0.83	0.30, 1.74	0.83
Occupant density, 10 residents/100 m²	0.97 [F]	0.87, 1.08 [F]	0.60	1.08 [F]	0.81, 1.44 [F]	0.58	0.77	0.38, 1.19	0.41
How many children 0-18 years-old live in house									
0	0.25	0.14, 0.43		1.42	0.36, 5.54		1.00	Reference	
1–3	0.21	0.10, 0.44	0.72	0.89	0.14, 5.80	0.69	0.43 [C]	0.00, 3.04	0.50

Characteristic	Air Pollutant								
	$PM_{2.5}$ barium, µg/m³			$PM_{2.5}$ brown carbon, µg/m³			$PM_{2.5}$ bromine, µg/m³		
	GM [A]	95% CI [A]	*p*-value [A]	GM [A]	95% CI [A]	*p*-value [A]	Exact OR [B]	Exact 95% CI [B]	Exact *p*-value [B]
How many children under 6 years-old live in house									
0	0.25	0.15, 0.43		1.43	0.37, 5.50		1.00	Reference	
1–3	0.15	0.064, 0.35	0.32	0.35	0.040, 3.03	0.27	0.54 [C]	0.00, 3.87	0.63
Primary fuel used for cooking									
Gas only	0.072	0.024, 0.22		0.15	0.014, 1.69		1.57	0.02, 36.60	
Wood only	0.25	0.15, 0.44		1.25	0.14, 10.87		1.00	Reference	
Other [G]	0.29	0.12, 0.73	0.11	1.80	0.49, 6.58	0.20	1.21 [C]	0.00, 10.78	1.00
Type of heating source in the home									
Electricity	0.13	0.076, 0.21		0.75	0.23, 2.49		1.00	Reference	
Other [H] or none	0.29	0.15, 0.56	0.05	2.79	0.54, 14.45	0.20	1.00	0.01, 22.49	1.00
Type of non-electric light source in the home									
Candle	0.19	0.10, 0.39		0.66	0.15, 2.94		0.84	0.04, 58.88	
Generator	0.30	0.12, 0.72		3.32	0.53, 20.75		0.75 [C]	0.00, 14.25	
Other [I] or none	0.13	0.045, 0.38	0.49	1.92	0.23, 16.16	0.38	1.00	Reference	0.57
Any smokers living in the home									
No	0.54	0.29, 1.00		2.07	0.34, 12.50		1.00	Reference	
Yes	0.13	0.081, 0.21	0.0004	0.71	0.19, 2.64	0.34	1.00	0.05, 65.47	1.00
How many smokers living in the home									
0	0.54	0.29, 1.00		2.10	0.36, 12.24		1.00	Reference	
1–2	0.12	0.053, 0.25		0.72	0.10, 5.47		1.25 [C]	0.00, 23.75	

Characteristic	PM2.5 barium, μg/m³			PM2.5 brown carbon, μg/m³			PM2.5 bromine, μg/m³		
	GM A	95% CI A	*p*-value A	GM A	95% CI A	*p*-value A	Exact OR B	Exact 95% CI B	Exact *p*-value B
3–4	0.20	0.089, 0.46	0.007 J	0.83	0.083, 8.30	0.69	1.73	0.02, 156.75	0.71
How many smokers living in the home regularly smoke inside the home									
0–1	0.11	0.055, 0.23		0.51	0.085, 3.11		1.00	Reference	
2–4	0.13	0.051, 0.33	0.81	1.07	0.11, 10.30	0.62	0.79 C	0.00, 7.05	0.86

Abbreviations: CI, confidence interval; GM, geometric mean; OR, odds ratio; PM2.5, particulate matter with an aerodynamic diameter less than 2.5 μm; A Estimated via Tobit regression models of the natural logarithm transformed values; B Estimated via exact unconditional logistic regression models; C Median unbiased estimate; D Using the Tukey-Kramer method to adjust for multiple comparisons, p-values for tests of pairwise differences among kilns were as follows: 1 vs. 2: 0.95, 1 vs. 3: 0.96, 1 vs. 4: 0.001, 2 vs. 3: 0.74, 2 vs. 4: 0.04, and 3 vs. 4: 0.002; E Unable to estimate; F Exponentiated regression coefficient and 95% CI (i.e., GM air pollutant concentration ratio for a specified change in the independent variable or exp(β) − 1 = percent change in GM air pollutant concentration for a specified change in the independent variable); G Includes coal and wood, gas and wood; H Includes lightbulb, line cable; I Includes candle and torch, fuel; J Using the Tukey-Kramer method to adjust for multiple comparisons, p-values for tests of pairwise differences among categories of how many smokers living in the home were as follows: 0 vs. 1–2: 0.007, 0 vs. 3–4: 0.15, and 1–2 vs. 3–4: 0.60.

Table S4. Associations between characteristics and $PM_{2.5}$ caesium, $PM_{2.5}$ calcium, and $PM_{2.5}$ chlorine air concentrations measured at on-site homes at brick kilns in Bhaktapur, Nepal, May 2018.

Characteristic	Air Pollutant								
	**$PM_{2.5}$ caesium, µg/m^3**			**$PM_{2.5}$ calcium, µg/m^3**			**$PM_{2.5}$ chlorine, µg/m^3**		
	**GM [A]**	**95% CI [A]**	***p*-value [A]**	**GM [B]**	**95% CI [B]**	***p*-value [B]**	**GM [A]**	**95% CI [A]**	***p*-value [A]**
Kiln number									
1	0.15	0.077, 0.30		1.83	0.98, 3.44		0.14	0.055, 0.36	
2	0.088	0.043, 0.18		1.13	0.60, 2.12		0.33	0.13, 0.81	
3	0.17	0.090, 0.33		1.54	0.82, 2.90		0.33	0.13, 0.81	
4	0.047	0.019, 0.11	0.08	0.35	0.19, 0.67	0.003[C]	0.15	0.060, 0.38	0.38
Type of home									
Worker	0.072	0.039, 0.13		0.74	0.44, 1.24		0.18	0.092, 0.34	
Fire master	0.14	0.086, 0.24	0.08	1.44	0.86, 2.42	0.07	0.27	0.14, 0.53	0.36
Location of sample									
Indoor	0.10	0.058, 0.18		1.19	0.69, 2.04		0.34	0.18, 0.65	
Outdoor	0.10	0.056, 0.18	0.98	0.90	0.52, 1.54	0.46	0.14	0.071, 0.27	0.05
Size of house, 50 feet^2	0.90[D]	0.49, 1.65[D]	0.74	1.23[D]	0.67, 2.25[D]	0.49	1.13[D]	0.53, 2.39[D]	0.75
Size of house, m^2	0.98[D]	0.86, 1.11[D]	0.73	1.04[D]	0.92, 1.19[D]	0.51	1.03[D]	0.87, 1.21[D]	0.75
How long lived in house, two months	1.10[D]	1.01, 1.20[D]	0.03	1.12[D]	1.02, 1.24[D]	0.02	0.86[D]	0.75, 0.99[D]	0.04
How many people live in house	1.04[D]	0.86, 1.26[D]	0.69	1.08[D]	0.87, 1.35[D]	0.46	0.90[D]	0.68, 1.20[D]	0.48
Occupant density, 10 residents/100 m^2	1.00[D]	0.91, 1.10[D]	>0.99	0.96[D]	0.87, 1.07[D]	0.47	1.02[D]	0.90, 1.16[D]	0.74
How many children 0-18 years-old live in house									
0	0.12	0.071, 0.19		1.21	0.72, 2.02		0.29	0.15, 0.54	
1–3	0.13	0.068, 0.25	0.78	1.09	0.54, 2.17	0.81	0.14	0.060, 0.33	0.19
How many children under 6 years-old live in house									
0	0.12	0.070, 0.19		1.21	0.72, 2.01		0.29	0.15, 0.54	
1–3	0.10	0.044, 0.21	0.68	0.83	0.38, 1.78	0.40	0.16	0.062, 0.41	0.31
Primary fuel used for cooking									
Gas only	0.042	0.014, 0.13		0.46	0.19, 1.08		0.10	0.037, 0.27	
Wood only	0.15	0.092, 0.23		1.26	0.76, 2.06		0.34	0.19, 0.60	
Other[E]	0.11	0.045, 0.25	0.13	1.42	0.60, 3.36	0.10	0.12	0.044, 0.32	0.05[F]
Type of heating source in the home									
Electricity	0.082	0.053, 0.13		0.60	0.39, 0.93		0.19	0.10, 0.36	

Characteristic	Air Pollutant								
	PM2.5 caesium, μg/m³			PM2.5 calcium, μg/m³			PM2.5 chlorine, μg/m³		
	GM A	95% CI A	*p*-value A	GM B	95% CI B	*p*-value B	GM A	95% CI A	*p*-value A
Other[G] or none	0.17	0.11, 0.27	0.02	1.66	0.91, 3.04	0.01	0.33	0.13, 0.82	0.33
Type of non-electric light source in the home									
Candle	0.095	0.048, 0.19		0.89	0.49, 1.62		0.14	0.045, 0.28	
Generator	0.14	0.065, 0.32		1.83	0.83, 4.07		0.14	0.059, 0.34	
Other[H] or none	0.10	0.038, 0.24	0.69	0.77	0.31, 1.93	0.26	0.57	0.22, 1.51	0.05
Any smokers living in the home									
No	0.21	0.12, 0.37		2.48	1.39, 4.45		0.15	0.063, 0.36	
Yes	0.082	0.051, 0.13	0.01	0.68	0.45, 1.03	0.001	0.25	0.14, 0.45	0.35
How many smokers living in the home									
0	0.21	0.12, 0.37		2.48	1.31, 4.71		0.16	0.077, 0.33	
1–2	0.092	0.047, 0.18		0.71	0.35, 1.45		0.14	0.064, 0.31	
3–4	0.12	0.056, 0.24	0.14	0.94	0.41, 2.15	0.03[I]	0.69	0.28, 1.71	0.02[J]
How many smokers living in the home regularly smoke inside the home									
0–1	0.089	0.054, 0.15		0.62	0.35, 1.11		0.24	0.11, 0.54	
2–4	0.10	0.050, 0.18	0.83	0.80	0.35, 1.82	0.60	0.36	0.12, 1.12	0.57

Abbreviations: CI, confidence interval; GM, geometric mean; PM2.5, particulate matter with an aerodynamic diameter less than 2.5 μm; A Estimated via Tobit regression models of the natural logarithm transformed values; B Estimated via linear regression models of the natural logarithm transformed values; C Using the Tukey method to adjust for multiple comparisons, p-values for tests of pairwise differences among kilns were as follows: 1 vs. 2: 0.68, 1 vs. 3: 0.98, 1 vs. 4: 0.004, 2 vs. 3: 0.89, 2 vs. 4: 0.06, and 3 vs. 4: 0.01; D Exponentiated regression coefficient and 95% CI (i.e., GM air pollutant concentration ratio for a specified change in the independent variable or exp(β) – 1 = percent change in GM air pollutant concentration for a specified change in the independent variable); E Includes coal and wood, gas and wood; F Using the Tukey-Kramer method to adjust for multiple comparisons, p-values for tests of pairwise differences among primary fuels used for cooking were as follows: Gas only vs. Wood only: 0.09, Gas only vs. Other: 0.97, and Wood only vs. Other: 0.17; G Includes lightbulb, line cable; H Includes candle and torch, fuel; I Using the Tukey method to adjust for multiple comparisons, p-values for tests of pairwise differences among categories of how many smokers living in the home were as follows: 0 vs. 1–2: 0.03, 0 vs. 3–4: 0.15, and 1–2 vs. 3–4: 0.85; J Using the Tukey-Kramer method to adjust for multiple comparisons, *p*-values for tests of pairwise differences among categories of how many smokers living in the home were as follows: 0 vs. 1–2: 0.97, 0 vs. 3–4: 0.03, and 1–2 vs. 3–4: 0.02.

Table S5. Associations between characteristics and $PM_{2.5}$ chromium, $PM_{2.5}$ cobalt, and $PM_{2.5}$ iron air concentrations measured at on-site homes at brick kilns in Bhaktapur, Nepal, May 2018.

Characteristic	Air Pollutant								
	$PM_{2.5}$ chromium, µg/m³			$PM_{2.5}$ cobalt, µg/m³			$PM_{2.5}$ iron, µg/m³		
	GM [A]	95% CI [A]	*p*-value [A]	GM [A]	95% CI [A]	*p*-value [A]	GM [B]	95% CI [B]	*p*-value [B]
Kiln number									
1	0.079	0.051, 0.12		0.014	0.0053, 0.039		4.81	2.30, 10.08	
2	0.051	0.028, 0.10		0.017	0.0069, 0.042		3.40	1.62, 7.12	
3	0.11	0.074, 0.15		0.018	0.0069, 0.049		6.60	3.15, 13.83	
4	0.053	0.029, 0.10	0.11	0.0029	0.00066, 0.013	0.17	0.88	0.41, 1.80	0.002[C]
Type of home									
Worker	0.058	0.036, 0.095		0.0072	0.0032, 0.016		2.18	1.17, 4.06	
Fire master	0.082	0.057, 0.12	0.20	0.019	0.096, 0.038	0.06	4.42	2.37, 8.24	0.11
Location of sample									
Indoor	0.056	0.035, 0.090		0.012	0.0057, 0.027		3.44	1.80, 6.56	
Outdoor	0.087	0.062, 0.12	0.08	0.011	0.0047, 0.025	0.81	2.80	1.47, 5.35	0.65
Size of house, 50 feet²	1.00 [D]	0.70, 1.43 [D]	0.99	1.27[D]	0.55, 2.96 [D]	0.57	1.25 [D]	0.61, 2.53[D]	0.53
Size of house, m²	1.00 [D]	0.92, 1.08 [D]	0.99	1.05[D]	0.88, 1.26 [D]	0.59	1.05 [D]	0.90, 1.22[D]	0.54
How long lived in house, two months	1.03 [D]	0.98, 1.09 [D]	0.23	1.17[D]	1.03, 1.32 [D]	0.01	1.13 [D]	1.01, 1.26[D]	0.04
How many people live in house	0.95 [D]	0.83, 1.07 [D]	0.37	1.04[D]	0.72, 1.48 [D]	0.84	0.99 [D]	0.77, 1.28[D]	0.94
Occupant density, 10 residents/100 m²	0.98 [D]	0.92, 1.04 [D]	0.48	0.96[D]	0.81, 1.14 [D]	0.62	0.94 [D]	0.84, 1.06[D]	0.30
How many children 0-18 years-old live in house									
0	0.073	0.051, 0.11		0.013	0.0057, 0.027		3.92	2.15, 7.14	
1–3	0.081	0.052, 0.12	0.71	0.010	0.0033, 0.030	0.73	3.14	1.40, 7.02	0.65
How many children under 6 years-old live in house									
0	0.071	0.048, 0.11		0.013	0.0058, 0.028		3.92	2.16, 7.14	
1–3	0.078	0.046, 0.13	0.76	0.0050	0.0012, 0.021	0.23	2.35	0.96, 5.77	0.34

Characteristic	Air Pollutant								
	PM2.5 chromium, μg/m³			PM2.5 cobalt, μg/m³			PM2.5 iron, μg/m³		
	GM [A]	95% CI [A]	*p*-value [A]	GM [A]	95% CI [A]	*p*-value [A]	GM [B]	95% CI [B]	*p*-value [B]
Primary fuel used for cooking									
Gas only	0.0026	[E]		0.0053	0.0012, 0.024		1.14	0.41, 3.14	
Wood only	0.086	0.064, 0.12		0.012	0.0053, 0.028		4.04	2.24, 7.26	
Other[F]	0.074	0.043, 0.13	0.87	0.012	0.0035, 0.044	0.59	4.29	1.55, 11.86	0.09
Type of heating source in the home									
Electricity	0.067	0.046, 0.10		0.0083	0.0040, 0.017		1.85	1.05, 3.26	
Other[G] or none	0.088	0.061, 0.13	0.22	0.014	0.0058, 0.034	0.34	4.34	1.94, 9.69	0.09
Type of non-electric light source in the home									
Candle	0.061	0.034, 0.11		0.012	0.0048, 0.031		2.92	1.40, 6.11	
Generator	0.072	0.034, 0.13		0.013	0.0038, 0.043		4.81	1.82, 12.77	
Other [H] or none	0.065	0.032, 0.13	0.90	0.0055	0.0012, 0.026	0.63	2.12	0.69, 6.54	0.51
Any smokers living in the home									
No	0.091	0.061, 0.14		0.032	0.015, 0.071		8.56	4.24, 17.27	
Yes	0.065	0.044, 0.10	0.19	0.0061	0.0028, 0.013	0.003	1.93	1.17, 3.17	0.001
How many smokers living in the home									
0	0.093	0.064, 0.13		0.032	0.014, 0.074		8.56	3.98, 18.39	
1-2	0.078	0.050, 0.12		0.0062	0.0018, 0.022		2.05	0.87, 4.82	
3-4	0.078	0.051, 0.13	0.77	0.0059	0.0015, 0.023	0.03[I]	2.54	0.95, 6.82	0.04[J]
How many smokers living in the home regularly smoke inside the home									

Characteristic	Air Pollutant								
	$PM_{2.5}$ chromium, μg/m³			$PM_{2.5}$ cobalt, μg/m³			$PM_{2.5}$ iron, μg/m³		
	**GM [A]**	**95% CI [A]**	***p*-value [A]**	**GM [A]**	**95% CI [A]**	***p*-value [A]**	**GM [B]**	**95% CI [B]**	***p*-value [B]**
0–1	0.076	0.051, 0.11		0.0049	0.0011, 0.022		1.89	0.91, 3.94	
2–4	0.071	0.039, 0.13	0.85	0.0027	0.00030, 0.025	0.58	1.89	0.67, 5.32	0.99

Abbreviations: CI, confidence interval; GM, geometric mean; PM2.5, particulate matter with an aerodynamic diameter less than 2.5 μm; A Estimated via Tobit regression models of the natural logarithm transformed values; B Estimated via linear regression models of the natural logarithm transformed values; C Using the Tukey method to adjust for multiple comparisons, p-values for tests of pairwise differences among kilns were as follows: 1 vs. 2: 0.90, 1 vs. 3: 0.93, 1 vs. 4: 0.01, 2 vs. 3: 0.57, 2 vs. 4: 0.05, and 3 vs. 4: 0.002; D Exponentiated regression coefficient and 95% CI (i.e., GM air pollutant concentration ratio for a specified change in the independent variable or exp(β) – 1 = percent change in GM air pollutant concentration for a specified change in the independent variable); E Unable to estimate; F Includes coal and wood, gas and wood; G Includes lightbulb, line cable; H Includes candle and torch, fuel; I Using the Tukey-Kramer method to adjust for multiple comparisons, p-values for tests of pairwise differences among categories of how many smokers living in the home were as follows: 0 vs. 1–2: 0.08, 0 vs. 3–4: 0.09, and 1–2 vs. 3–4: > 0.99; J Using the Tukey method to adjust for multiple comparisons, p-values for tests of pairwise differences among categories of how many smokers living in the home were as follows: 0 vs. 1–2: 0.04, 0 vs. 3–4: 0.13, and 1–2 vs. 3–4: 0.94.

Table S6. Associations between characteristics and $PM_{2.5}$ lead, $PM_{2.5}$ magnesium, and $PM_{2.5}$ manganese air concentrations measured at on-site homes at brick kilns in Bhaktapur, Nepal, May 2018.

Characteristic	Air Pollutant								
	$PM_{2.5}$ lead, µg/m³			$PM_{2.5}$ magnesium, µg/m³			$PM_{2.5}$ manganese, µg/m³		
	GM [A]	95% CI [A]	*p*-value [A]	GM [A]	95% CI [A]	*p*-value [A]	GM [A]	95% CI [A]	*p*-value [A]
Kiln number									
1	0.0087	0.0029, 0.026		0.14	0.096, 0.20		0.055	0.027, 0.11	
2	0.019	0.0092, 0.041		0.12	0.084, 0.17		0.032	0.015, 0.066	
3	0.040	0.021, 0.076		0.0064	[B]		0.073	0.036, 0.15	
4	0.0078	0.0026, 0.024	0.03[C]	0.11	0.074, 0.16	0.79	0.0097	0.0038, 0.025	0.01[D]
Type of home									
Worker	0.011	0.0049, 0.026		0.11	0.076, 0.15		0.027	0.015, 0.050	
Fire master	0.020	0.010, 0.040	0.23	0.087	0.057, 0.13	0.36	0.045	0.024, 0.081	0.26
Location of sample									
Indoor	0.012	0.0050, 0.027		0.11	0.077, 0.15		0.039	0.021, 0.072	
Outdoor	0.019	0.0095, 0.038	0.32	0.087	0.057, 0.13	0.33	0.031	0.016, 0.058	0.62
Size of house, 50 feet²	1.08[E]	0.57, 2.04[E]	0.82	1.06[E]	0.79, 1.42[E]	0.69	1.12[E]	0.57, 2.21[E]	0.74
Size of house, m²	1.02[E]	0.88, 1.17[E]	0.83	1.01[E]	0.95, 1.08[E]	0.70	1.02[E]	0.88, 1.19[E]	0.75
How long lived in house, two months	0.98[E]	0.84, 1.13[E]	0.75	1.00[E]	0.92, 1.09[E]	>0.99	1.13[E]	1.02, 1.24[E]	0.02
How many people live in house	0.82[E]	0.62, 1.07[E]	0.14	1.09[E]	0.94, 1.25[E]	0.26	0.96[E]	0.76, 1.21[E]	0.73
Occupant density, 10 residents/100 m²	0.91[E]	0.77, 1.07[E]	0.23	1.02[E]	0.95, 1.09[E]	0.57	0.93[E]	0.83, 1.05[E]	0.26
How many children 0-18 years-old live in house									
0	0.014	0.0062, 0.034		0.074	0.042, 0.13		0.042	0.025, 0.073	
1–3	0.012	0.0040, 0.036	0.75	0.10	0.056, 0.18	0.35	0.038	0.018, 0.080	0.83

Characteristic	Air Pollutant								
	PM2.5 lead, μg/m³			PM2.5 magnesium, μg/m³			PM2.5 manganese, μg/m³		
	GM [A]	95% CI [A]	*p*-value [A]	GM [A]	95% CI [A]	*p*-value [A]	GM [A]	95% CI [A]	*p*-value [A]
How many children under 6 years-old live in house									
0	0.013	0.0053, 0.034		0.075	0.044, 0.13		0.042	0.024, 0.074	
1–3	0.0099	0.0025, 0.040	0.67	0.11	0.064, 0.20	0.22	0.030	0.013, 0.072	0.53
Primary fuel used for cooking									
Gas only	0.000020	[B]		0.096	0.056, 0.17		0.014	0.0046, 0.043	
Wood only	0.024	0.014, 0.043		0.070	0.041, 0.12		0.042	0.024, 0.074	
Other [F]	0.000020	[B]	> 0.99	0.15	0.097, 0.24	0.07	0.050	0.020, 0.13	0.17
Type of heating source in the home									
Electricity	0.013	0.0058, 0.030		0.093	0.062, 0.14		0.024	0.014, 0.041	
Other [G] or none	0.014	0.0046, 0.042	0.93	0.11	0.064, 0.18	0.59	0.044	0.021, 0.092	0.19
Type of non-electric light source in the home									
Candle	0.016	0.0072, 0.034		0.088	0.057, 0.14		0.036	0.018, 0.071	
Generator	0.0068	0.0017, 0.028		0.13	0.081, 0.20		0.054	0.023, 0.13	
Other [H] or none	0.016	0.0054, 0.050	0.47	0.074	0.036, 0.15	0.26	0.019	0.0064, 0.058	0.35
Any smokers living in the home									
No	0.014	0.0045, 0.041		0.062	0.029, 0.13		0.087	0.046, 0.17	
Yes	0.012	0.0048, 0.029	0.80	0.098	0.065, 0.15	0.21	0.023	0.014, 0.038	0.001
How many smokers living in the home									
0	0.012	0.0033, 0.044		0.048	0.015, 0.15		0.087	0.044, 0.17	

Characteristic	Air Pollutant								
	PM2.5 lead, μg/m³			PM2.5 magnesium, μg/m³			PM2.5 manganese, μg/m³		
	GM [A]	95% CI [A]	*p*-value [A]	GM [A]	95% CI [A]	*p*-value [A]	GM [A]	95% CI [A]	*p*-value [A]
1–2	0.012	0.0033, 0.047		0.077	0.032, 0.19		0.026	0.012, 0.059	
3–4	0.0079	0.0012, 0.051	0.90	0.087	0.036, 0.21	0.58	0.026	0.0099, 0.068	0.04 [I]
How many smokers living in the home regularly smoke inside the home									
0–1	0.023	0.012, 0.044		0.076	0.042, 0.14		0.022	0.010, 0.050	
2–4	0.000040	[B]	> 0.99	0.14	0.086, 0.22	0.07	0.020	0.0064, 0.062	0.86

Abbreviations: CI, confidence interval; GM, geometric mean; PM2.5, particulate matter with an aerodynamic diameter less than 2.5 μm; A Estimated via Tobit regression models of the natural logarithm transformed values; B Unable to estimate; C Using the Tukey-Kramer method to adjust for multiple comparisons, p-values for tests of pairwise differences among kilns were as follows: 1 vs. 2: 0.57, 1 vs. 3: 0.07, 1 vs. 4: > 0.99, 2 vs. 3: 0.45, 2 vs. 4: 0.47, and 3 vs. 4: 0.05; D Using the Tukey-Kramer method to adjust for multiple comparisons, p-values for tests of pairwise differences among kilns were as follows: 1 vs. 2: 0.71, 1 vs. 3: 0.95, 1 vs. 4: 0.02, 2 vs. 3: 0.38, 2 vs. 4: 0.20, and 3 vs. 4: 0.004; E Exponentiated regression coefficient and 95% CI (i.e., GM air pollutant concentration ratio for a specified change in the independent variable or exp(β) – 1 = percent change in GM air pollutant concentration for a specified change in the independent variable); F Includes coal and wood, gas and wood; G Includes lightbulb, line cable; H Includes candle and torch, fuel; I Using the Tukey-Kramer method to adjust for multiple comparisons, p-values for tests of pairwise differences among categories of how many smokers living in the home were as follows: 0 vs. 1–2: 0.07, 0 vs. 3–4: 0.11, and 1–2 vs. 3–4: 1.00.

Table S7. Associations between characteristics and $PM_{2.5}$ nickel, $PM_{2.5}$ potassium, and $PM_{2.5}$ rubidium air concentrations measured at on-site homes at brick kilns in Bhaktapur, Nepal, May 2018.

Characteristic	Air Pollutant								
	$PM_{2.5}$ nickel, μg/m^3			$PM_{2.5}$ potassium, μg/m^3			$PM_{2.5}$ rubidium, μg/m^3		
	Exact OR[A]	Exact 95% CI [A]	Exact *p*-value [A]	GM [B]	95% CI [B]	*p*-value [B]	GM [C]	95% CI [C]	*p*-value [C]
Kiln number									
1	1.00	Reference		2.09	1.15, 3.80		0.012	0.0045, 0.033	
2	0.16	< 0.01, 2.42		2.28	1.26, 4.15		0.011	0.0041, 0.030	
3	0.62	0.05, 6.42		4.65	2.56, 8.45		0.026	0.011, 0.061	
4	0.16	< 0.01, 2.42	0.34	0.59	0.32, 1.06	0.0003[D]	0.0000078	[E]	0.53
Type of home									
Worker	1.00	Reference		1.43	0.83, 2.47		0.0073	0.0026, 0.020	
Fire master	2.52	0.41, 19.57	0.43	2.52	1.46, 4.35	0.14	0.014	0.0061, 0.031	0.26
Location of sample									
Indoor	1.00	Reference		2.09	1.19, 3.66		0.012	0.0051, 0.028	
Outdoor	1.35	0.22, 8.74	1.00	1.73	0.98, 3.04	0.63	0.0082	0.0030, 0.023	0.54
Size of house, 50 feet^2	0.88	0.28, 2.77	0.84	1.17[F]	0.63, 2.15[F]	0.61	0.74[F]	0.32, 1.75[F]	0.50
Size of house, m^2	0.97	0.75, 1.25	0.84	1.03[F]	0.90, 1.18[F]	0.62	0.94[F]	0.78, 1.13[F]	0.48
How long lived in house, two months	1.20	0.97, 1.52	0.10	1.08[F]	0.97, 1.19[F]	0.15	1.15[F]	1.02, 1.30[F]	0.02
How many people live in house	1.16	0.77, 1.76	0.50	0.90[F]	0.72, 1.12[F]	0.32	0.83[F]	0.58, 1.18[F]	0.30
Occupant density, 10 residents/100 m^2	1.02	0.84, 1.21	0.83	0.94[F]	0.85, 1.03[F]	0.17	0.96[F]	0.81, 1.13[F]	0.60

Characteristic	Air Pollutant								
	PM2.5 nickel, µg/m³			PM2.5 potassium, µg/m³			PM2.5 rubidium, µg/m³		
	Exact OR[A]	Exact 95% CI [A]	Exact *p*-value [A]	GM [B]	95% CI [B]	*p*-value [B]	GM [C]	95% CI [C]	*p*-value [C]
How many children 0-18 years-old live in house									
0	1.00	Reference		2.54	1.53, 4.22		0.011	0.0048, 0.027	
1-3	1.70	0.24, 11.63	0.80	1.61	0.82, 3.18	0.28	0.0089	0.0027, 0.029	0.71
How many children under 6 years-old live in house									
0	1.00	Reference		2.54	1.52, 4.27		0.011	0.0043, 0.027	
1-3	0.87	0.07, 7.57	1.00	1.35	0.62, 2.93	0.17	0.0074	0.0017, 0.032	0.62
Primary fuel used for cooking									
Gas only	0.41	0.01, 5.11		0.73	0.31, 1.73		0.0042	0.00073, 0.025	
Wood only	1.00	Reference		2.54	1.54, 4.19		0.012	0.0053, 0.029	
Other [G]	1.00	0.07, 9.65	0.87	2.15	0.91, 5.12	0.05[H]	0.0093	0.0023, 0.038	0.49
Type of heating source in the home									
Electricity	1.00	Reference		1.28	0.75, 2.19		0.0074	0.0026, 0.021	
Other[I] or none	2.49	0.25, 25.87	0.60	2.31	1.09, 4.90	0.20	0.013	0.0050, 0.035	0.31
Type of non-electric light source in the home									
Candle	1.94	0.13, 117.90		1.88	0.98, 3.61		0.011	0.0039, 0.032	
Generator	4.45	0.28, 294.49		2.09	0.88, 4.94		0.0097	0.0028, 0.036	
Other [J] or none	1.00	Reference	0.45	1.54	0.57, 4.16	0.89	0.0043	0.00065, 0.029	0.63
Any smokers living in the home									
No	1.00	Reference		3.89	2.03, 7.48		0.020	0.0082, 0.050	

Characteristic	Air Pollutant								
	$PM_{2.5}$ nickel, μg/m³			$PM_{2.5}$ potassium, μg/m³			$PM_{2.5}$ rubidium, μg/m³		
	Exact OR [A]	Exact 95% CI [A]	Exact *p*-value [A]	GM [B]	95% CI [B]	*p*-value [B]	GM [C]	95% CI [C]	*p*-value [C]
Yes	0.13	0.01, 0.90	0.04	1.34	0.85, 2.13	0.01	0.0062	0.0022, 0.017	0.06
How many smokers living in the home									
0	1.00	Reference		3.89	2.02, 7.51		0.020	0.0081, 0.051	
1–2	0.24	0.02, 2.35		1.16	0.56, 2.42		0.0047	0.00092, 0.024	
3–4	0.15	< 0.01, 2.14	0.16	1.87	0.80, 4.37	0.06	0.010	0.0025, 0.039	0.24
How many smokers living in the home regularly smoke inside the home									
0–1	1.00	Reference		1.45	0.71, 2.97		0.0076	0.0019, 0.030	
2–4	1.00	0.01, 23.97	1.00	1.14	0.41, 3.14	0.68	0.0056	0.00080, 0.039	0.74

Abbreviations: CI, confidence interval; GM, geometric mean; OR, odds ratio; $PM_{2.5}$, particulate matter with an aerodynamic diameter less than 2.5 μm; [A] Estimated via exact unconditional logistic regression models; [B] Estimated via linear regression models of the natural logarithm transformed values; [C] Estimated via Tobit regression models of the natural logarithm transformed values; [D] Using the Tukey method to adjust for multiple comparisons, p-values for tests of pairwise differences among kilns were as follows: 1 vs. 2: > 0.99, 1 vs. 3: 0.24, 1 vs. 4: 0.02, 2 vs. 3: 0.33, 2 vs. 4: 0.01, and 3 vs. 4: 0.0001; [E] Unable to estimate; [F] Exponentiated regression coefficient and 95% CI (i.e., GM air pollutant concentration ratio for a specified change in the independent variable or $\exp(\beta) - 1$ = percent change in GM air pollutant concentration for a specified change in the independent variable); [G] Includes coal and wood, gas and wood; [H] Using the Tukey method to adjust for multiple comparisons, p-values for tests of pairwise differences among primary fuels used for cooking were as follows: Gas only vs. Wood only: 0.04, Gas only vs. Other: 0.18, and Wood only vs. Other: 0.94; [I] Includes lightbulb, line cable; [J] Includes candle and torch, fuel.

Table S8. Associations between characteristics and $PM_{2.5}$ silicon, $PM_{2.5}$ sodium, and $PM_{2.5}$ strontium air concentrations measured at on-site homes at brick kilns in Bhaktapur, Nepal, May 2018.

Characteristic	Air Pollutant								
	$PM_{2.5}$ silicon, μg/m³			$PM_{2.5}$ sodium, μg/m³			$PM_{2.5}$ strontium, μg/m³		
	GM [A]	95% CI [A]	*p*-value [A]	Exact OR [B]	Exact 95% CI[B]	Exact *p*-value [B]	Exact OR[B]	Exact 95% CI[B]	Exact *p*-value [B]
Kiln number									
1	12.22	6.20, 24.09		1.00	Reference		1.00	Reference	
2	8.75	4.44, 17.26		7.92[C]	1.14, ∞		0.26	<0.01, 4.40	
3	15.48	7.85, 30.53		1.00[C]	0.05, ∞		1.61	0.16, 18.77	
4	2.47	1.25, 4.87	0.003[D]	[E]	[E]	0.03	0.21[C]	0.00, 1.53	0.12
Type of home									
Worker	6.10	3.42, 10.85		1.00	Reference		1.00	Reference	
Fire master	10.49	5.89, 18.68	0.18	4.77	0.40, 262.30	0.33	4.01	0.56, 48.74	0.22
Location of sample									
Indoor	8.71	4.82, 15.75		1.00	Reference		1.00	Reference	
Outdoor	7.34	4.06, 13.28	0.68	0.63	0.05, 6.43	1.00	0.25	0.02, 1.78	0.22
Size of house, 50 feet²	1.19[F]	0.62, 2.26[F]	0.59	6.58	1.23, 64.73	0.03	0.68	0.20, 2.15	0.53
Size of house, m²	1.04[F]	0.90, 1.19[F]	0.60	1.50	1.04, 2.46	0.03	0.92	0.71, 1.18	0.52
How long lived in house, two months	1.12[F]	1.00, 1.24[F]	0.05	0.87	0.45, 1.27	0.94	1.23	1.00, 1.61	0.05
How many people live in house	0.99[F]	0.78, 1.25[F]	0.94	0.83	0.37, 1.52	0.68	0.98	0.63, 1.48	0.98
Occupant density, 10 residents/100 m²	0.95[F]	0.85, 1.06[F]	0.32	0.54	0.21, 1.00	0.05	0.99	0.80, 1.18	0.97
How many children 0-18 years-old live in house									

Characteristic	Air Pollutant								
	PM2.5 silicon, µg/m³			PM2.5 sodium, µg/m³			PM2.5 strontium, µg/m³		
	GM [A]	95% CI [A]	*p*-value [A]	Exact OR [B]	Exact 95% CI[B]	Exact *p*-value [B]	Exact OR[B]	Exact 95% CI[B]	Exact *p*-value [B]
0	9.70	5.54, 16.99		1.00	Reference		1.00	Reference	
1-3	7.96	3.75, 16.87	0.67	0.43[C]	0.00, 3.04	0.50	1.11	0.13, 7.96	1.00
How many children under 6 years-old live in house									
0	9.70	5.53, 17.03		1.00	Reference		1.00	Reference	
1-3	6.15	2.64, 14.31	0.36	0.54[C]	0.00, 3.87	0.63	0.38	0.01, 4.55	0.76
Primary fuel used for cooking									
Gas only	3.52	1.37, 9.10		1.21[C]	0.00, 10.78		0.41	0.01, 5.11	
Wood only	9.71	5.62, 16.79		1.00	Reference		1.00	Reference	
Other[G]	11.17	4.33, 28.83	0.14	1.57	0.02, 36.60	1.00	0.41	0.01, 5.11	0.62
Type of heating source in the home									
Electricity	5.06	2.99, 8.57		1.00	Reference		1.00	Reference	
Other[H] or none	10.86	5.16, 22.84	0.10	2.07	0.02, 179.22	1.00	2.25	0.13, 38.09	0.81
Type of non-electric light source in the home									
Candle	7.87	4.02, 15.39		0.41	<0.01, 36.18		2.72[C]	0.40, ∞	
Generator	12.22	5.03, 29.71		0.75[C]	0.00, 14.25		3.62[C]	0.48, ∞	
Other[I] or none	5.20	1.87, 14.51	0.44	1.00	Reference	0.70	1.00	Reference	0.38
Any smokers living in the home									
No	20.06	10.51, 38.26		1.00	Reference		1.00	Reference	
Yes	5.20	3.29, 8.21	0.002	1.00	0.05, 65.47	1.00	0.03	<0.01, 0.32	0.0008
How many smokers living in the home									

Characteristic	Air Pollutant								
	PM2.5 silicon, μg/m³			PM2.5 sodium, μg/m³			PM2.5 strontium, μg/m³		
	GM [A]	95% CI [A]	*p*-value [A]	Exact OR [B]	Exact 95% CI[B]	Exact *p*-value [B]	Exact OR[B]	Exact 95% CI[B]	Exact *p*-value [B]
0	20.06	9.80, 41.03		1.00	Reference		1.00	Reference	
1-2	5.13	2.30, 11.41		1.25[C]	0.00, 23.75		0.07	<0.01, 0.97	
3-4	6.63	2.63, 16.71	0.03[J]	1.73	0.02, 156.75	0.71	0.08[C]	0.00, 0.51	0.005
How many smokers living in the home regularly smoke inside the home									
0-1	5.09	2.59, 9.99		1.00	Reference		1.00	Reference	
2-4	5.19	2.00, 13.48	0.97	2.00[C]	0.00, 38.00	1.00	2.00[C]	0.00, 38.00	1.00

Abbreviations: CI, confidence interval; GM, geometric mean; OR, odds ratio; $PM_{2.5}$, particulate matter with an aerodynamic diameter less than 2.5 μm; [A] Estimated via linear regression models of the natural logarithm transformed values; [B] Estimated via exact unconditional logistic regression models; [C] Median unbiased estimate; [D] Using the Tukey method to adjust for multiple comparisons, *p*-values for tests of pairwise differences among kilns were as follows: 1 vs. 2: 0.89, 1 vs. 3: 0.96, 1 vs. 4: 0.01, 2 vs. 3: 0.62, 2 vs. 4: 0.05, and 3 vs. 4: 0.003; [E] Unable to estimate; [F] Exponentiated regression coefficient and 95% CI (i.e., GM air pollutant concentration ratio for a specified change in the independent variable or $\exp(\beta) - 1$ = percent change in GM air pollutant concentration for a specified change in the independent variable); [G] Includes coal and wood, gas and wood; [H] Includes lightbulb, line cable; [I] Includes candle and torch, fuel; [J] Using the Tukey method to adjust for multiple comparisons, p-values for tests of pairwise differences among categories of how many smokers living in the home were as follows: 0 vs. 1-2: 0.04, 0 vs. 3-4: 0.14, and 1-2 vs. 3-4: 0.90.

Table S9. Associations between characteristics and PM2.5 sulfur, PM2.5 titanium, and PM2.5 vanadium air concentrations measured at on-site homes at brick kilns in Bhaktapur, Nepal, May 2018.

Characteristic	Air Pollutant								
	PM2.5 sulfur, μg/m³			PM2.5 titanium, μg/m³			PM2.5 vanadium, μg/m³		
	GM A	95% CI A	*p*-value A	GM B	95% CI B	*p*-value B	Exact OR C	Exact 95% CI C	Exact *p*-value C
Kiln number									
1	1.55	1.18, 2.04		0.56	0.26, 1.23		1.00	Reference	
2	2.42	1.84, 3.19		0.37	0.17, 0.82		0.38[D]	0.00, 3.36	
3	4.29	3.26, 5.65		0.85	0.39, 1.87		1.73	0.14, 28.99	
4	1.44	1.10, 1.89	< 0.0001[E]	0.080	0.035, 0.18	0.0003[F]	0.38[D]	0.00, 3.36	0.13
Type of home									
Worker	1.97	1.48, 2.64		0.23	0.12, 0.45		1.00	Reference	
Fire master	2.45	1.83, 3.27	0.29	0.53	0.27, 1.04	0.08	4.77	0.40, 262.30	0.33
Location of sample									
Indoor	2.15	1.60, 2.88		0.37	0.18, 0.75		1.00	Reference	
Outdoor	2.25	1.67, 3.02	0.82	0.33	0.16, 0.67	0.81	0.21	<0.01, 2.49	0.33
Size of house, 50 feet²	1.12[G]	0.82, 1.53[G]	0.46	1.31[G]	0.60, 2.86[G]	0.50	0.35	0.06, 1.49	0.17
Size of house, m²	1.02[G]	0.96, 1.10[G]	0.47	1.06[G]	0.89, 1.25[G]	0.51	0.79	0.55, 1.09	0.16
How long lived in house, two months	1.04[G]	0.98, 1.10[G]	0.18	1.14[G]	1.01, 1.30[G]	0.04	1.36	1.06, 1.82	0.01
How many people live in house	0.89[G]	0.79, 0.99[G]	0.04	1.01[G]	0.76, 1.34[G]	0.97	0.95	0.56, 1.49	0.87
Occupant density, 10 residents/100 m²	0.93[G]	0.89, 0.98[G]	0.01	0.93[G]	0.81, 1.06[G]	0.27	1.02	0.82, 1.23	0.79
How many children 0-18 years-old live in house									
0	2.66	2.02, 3.50		0.45	0.23, 0.88		1.00	Reference	
1-3	1.75	1.21, 2.53	0.07	0.32	0.13, 0.79	0.55	1.24	0.09, 13.37	1.00
How many children under 6 years-old live in house									
0	2.66	2.00, 3.53		0.45	0.24, 0.88		1.00	Reference	
1-3	1.66	1.09, 2.55	0.07	0.21	0.078, 0.58	0.22	0.72	0.01, 11.06	1.00

Characteristic	Air Pollutant								
	PM2.5 sulfur, μg/m³			PM2.5 titanium, μg/m³			PM2.5 vanadium, μg/m³		
	GM [A]	95% CI [A]	*p*-value [A]	GM [B]	95% CI [B]	*p*-value [B]	Exact OR [C]	Exact 95% CI [C]	Exact *p*-value [C]
Primary fuel used for cooking									
Gas only	1.57	0.99, 2.51		0.11	0.034, 0.33		0.50[D]	0.00, 3.37	
Wood only	2.66	2.03, 3.49		0.49	0.26, 0.92		1.00	Reference	
Other[H]	1.76	1.10, 2.80	0.09	0.44	0.15, 1.31	0.06	0.71	0.01, 9.95	0.79
Type of heating source in the home									
Electricity	2.12	1.63, 2.76		0.20	0.11, 0.37		1.00	Reference	
Other[I] or none	1.75	1.20, 2.53	0.39	0.51	0.22, 1.19	0.08	2.07	0.02, 179.22	1.00
Type of non-electric light source in the home									
Candle	2.67	1.95, 3.65		0.33	0.15, 0.72		1.83[D]	0.25, ∞	
Generator	1.55	1.03, 2.35		0.56	0.20, 1.58		1.98[D]	0.22, ∞	
Other[J] or none	1.97	1.22, 3.18	0.11	0.22	0.066, 0.75	0.51	1.00	Reference	0.56
Any smokers living in the home									
No	2.92	2.03, 4.21		1.13	0.54, 2.38		1.00	Reference	
Yes	1.91	1.48, 2.48	0.06	0.20	0.12, 0.35	0.0002	0.09	<0.01, 1.10	0.06
How many smokers living in the home									
0	2.92	1.99, 4.30		1.13	0.50, 2.55		1.00	Reference	
1-2	2.05	1.33, 3.15		0.20	0.079, 0.51		0.23	<0.01, 3.26	
3-4	1.50	0.91, 2.46	0.10	0.25	0.087, 0.74	0.01[K]	0.24[D]	0.00, 1.64	0.20
How many smokers living in the home regularly smoke inside the home									
0-1	2.38	1.79, 3.16		0.21	0.097, 0.44		1.00	Reference	
2-4	1.15	0.77, 1.72	0.007	0.18	0.062, 0.53	0.84	2.00[D]	0.00, 38.00	1.00

Abbreviations: CI, confidence interval; GM, geometric mean; OR, odds ratio; $PM_{2.5}$, particulate matter with an aerodynamic diameter less than 2.5 μm; [A] Estimated via linear regression models of the natural logarithm transformed values; [B] Estimated via Tobit regression models of the natural logarithm transformed values; [C] Estimated via exact unconditional logistic regression models; [D] Median unbiased estimate; [E] Using the Tukey method to adjust for multiple comparisons, p-values for tests of pairwise

differences among kilns were as follows: 1 vs. 2: 0.11, 1 vs. 3: <0.0001, 1 vs. 4: 0.98, 2 vs. 3: 0.03, 2 vs. 4: 0.05, and 3 vs. 4: <0.0001; [F] Using the Tukey-Kramer method to adjust for multiple comparisons, p-values for tests of pairwise differences among kilns were as follows: 1 vs. 2: 0.89, 1 vs. 3: 0.88, 1 vs. 4: 0.005, 2 vs. 3: 0.46, 2 vs. 4: 0.04, and 3 vs. 4: 0.0003; [G] Exponentiated regression coefficient and 95% CI (i.e., GM air pollutant concentration ratio for a specified change in the independent variable or $\exp(\beta) - 1$ = percent change in GM air pollutant concentration for a specified change in the independent variable); [H] Includes coal and wood, gas and wood; [I] Includes lightbulb, line cable; [J] Includes candle and torch, fuel; [K] Using the Tukey-Kramer method to adjust for multiple comparisons, p-values for tests of pairwise differences among categories of how many smokers living in the home were as follows: 0 vs. 1-2: 0.02, 0 vs. 3-4: 0.07, and 1-2 vs. 3-4: 0.95.

Table S10. Associations between characteristics and $PM_{2.5}$ zinc air concentrations, relative humidity, and temperature measured at on-site homes at brick kilns in Bhaktapur, Nepal, May 2018.

Characteristic	Air Pollutant or Weather Variable								
	$PM_{2.5}$ zinc, μg/m³			Relative humidity, %			Temperature, °C		
	GM[A]	95% CI [A]	*p*-value [A]	GM [B]	95% CI [B]	*p*-value [B]	GM [B]	95% CI [B]	*p*-value [B]
Kiln number									
1	0.044	0.025, 0.078		22.56	20.07, 25.36		31.51	29.70, 33.43	
2	0.067	0.038, 0.12		35.36	31.46, 39.75		26.50	24.98, 28.12	
3	0.11	0.064, 0.20		53.69	47.38, 60.84		27.03	25.37, 28.79	
4	0.012	0.0062, 0.024	<0.0001[C]	65.13	57.94, 73.21	<0.0001[D]	22.10	20.84, 23.45	<0.0001[E]
Type of home									
Worker	0.037	0.022, 0.064		42.31	33.35, 53.69		25.67	23.72, 27.78	
Fire master	0.057	0.033, 0.099	0.27	38.89	30.88, 48.97	0.61	27.43	25.41, 29.62	0.23
Location of sample									
Indoor	0.049	0.028, 0.085		42.99	34.18, 54.07		26.25	24.27, 28.39	
Outdoor	0.044	0.025, 0.077	0.80	38.02	30.00, 48.19	0.45	26.90	24.81, 29.17	0.66
Size of house, 50 feet²	1.17[F]	0.62, 2.23[F]	0.63	0.82[F]	0.65, 1.04[F]	0.11	1.04[F]	0.96, 1.14[F]	0.30
Size of house, m²	1.03[F]	0.90, 1.19[F]	0.64	0.96[F]	0.91, 1.01[F]	0.11	1.01[F]	0.99, 1.03[F]	0.31
How long lived in house, two months	1.08[F]	0.97, 1.19[F]	0.15	1.00[F]	0.95, 1.05[F]	0.95	1.00[F]	0.99, 1.02[F]	0.76
How many people live in house	0.92[F]	0.74, 1.15[F]	0.45	0.87[F]	0.80, 0.93[F]	0.0005	1.03[F]	1.00, 1.06[F]	0.04
Occupant density, 10 residents/100 m²	0.90[F]	0.80, 1.01[F]	0.07	1.01[F]	0.96, 1.05[F]	0.81	1.00[F]	0.98, 1.01[F]	0.55
How many children 0-18 years-old live in house									
0	0.055	0.033, 0.093		43.05	34.73, 53.37		26.55	24.73, 28.51	
1-3	0.042	0.021, 0.085	0.55	32.72	24.15, 44.34	0.14	28.16	25.46, 31.15	0.34
How many children under 6 years-old live in house									
0	0.055	0.032, 0.094		43.05	35.16, 52.72		26.55	24.89, 28.32	
1-3	0.037	0.016, 0.081	0.41	38.51	27.83, 53.28	0.55	26.50	23.90, 29.39	0.97
Primary fuel used for cooking									
Gas only	0.019	0.0077, 0.046		54.84	38.96, 77.19		22.62	20.22, 25.32	
Wood only	0.044	0.019, 0.10		42.62	34.79, 52.22		27.28	25.52, 29.17	
Other[G]	0.062	0.038, 0.10	0.07	26.87	19.09, 37.82	0.02[H]	29.11	26.01, 32.57	0.01[I]
Type of heating source in the home									
Electricity	0.031	0.017, 0.054		52.92	45.07, 62.14		24.12	22.58, 25.76	

Characteristic	Air Pollutant or Weather Variable								
	PM2.5 zinc, μg/m³			Relative humidity, %			Temperature, °C		
	GM[A]	95% CI [A]	*p*-value [A]	GM [B]	95% CI [B]	*p*-value [B]	GM [B]	95% CI [B]	*p*-value [B]
Other[J] or none	0.059	0.027, 0.13	0.18	26.20	21.03, 32.64	<0.0001	30.26	27.65, 33.12	0.0004
Type of non-electric light source in the home									
Candle	0.051	0.027, 0.096		51.18	43.36, 60.42		24.65	23.03, 26.39	
Generator	0.044	0.020, 0.10		22.56	18.26, 27.87		31.51	28.89, 34.37	
Other[K] or none	0.032	0.011, 0.088	0.73	51.45	40.30, 65.67	<0.0001[L]	24.70	22.35, 27.31	0.0003[M]
Any smokers living in the home									
No	0.077	0.039, 0.15		32.65	24.58, 43.35		29.35	26.78, 32.16	
Yes	0.034	0.021, 0.057	0.06	45.91	37.37, 56.39	0.06	25.26	23.64, 26.99	0.01
How many smokers living in the home									
0	0.077	0.038, 0.16		32.65	23.89, 44.61		29.35	26.67, 32.29	
1-2	0.032	0.014, 0.074		47.07	32.41, 68.36		25.07	22.36, 28.10	
3-4	0.038	0.015, 0.099	0.25	40.70	27.20, 60.91	0.30	26.36	23.30, 29.82	0.10
How many smokers living in the home regularly smoke inside the home									
0-1	0.043	0.020, 0.092		55.10	43.77, 69.37		24.20	22.03, 26.60	
2-4	0.018	0.0060, 0.054	0.19	36.12	26.45, 49.34	0.03	27.08	23.84, 30.77	0.15

Abbreviations: CI, confidence interval; GM, geometric mean; PM2.5, particulate matter with an aerodynamic diameter less than 2.5 μm; [A] Estimated via Tobit regression models of the natural logarithm transformed values; [B] Estimated via linear regression models of the natural logarithm transformed values; [C] Using the Tukey-Kramer method to adjust for multiple comparisons, p-values for tests of pairwise differences among kilns were as follows: 1 vs. 2: 0.74, 1 vs. 3: 0.10, 1 vs. 4: 0.02, 2 vs. 3: 0.58, 2 vs. 4: 0.0009, and 3 vs. 4: < 0.0001; [D] Using the Tukey method to adjust for multiple comparisons, p-values for tests of pairwise differences among kilns were as follows: 1 vs. 2: < 0.0001, 1 vs. 3: < 0.0001, 1 vs. 4: < 0.0001, 2 vs. 3: 0.0002, 2 vs. 4: < 0.0001, and 3 vs. 4: 0.12; [E] Using the Tukey method to adjust for multiple comparisons, p-values for tests of pairwise differences among kilns were as follows: 1 vs. 2: 0.001, 1 vs. 3: 0.006, 1 vs. 4: < 0.0001, 2 vs. 3: 0.97, 2 vs. 4: 0.0007, and 3 vs. 4: 0.0003; [F] Exponentiated regression coefficient and 95% CI (i.e., GM air pollutant concentration or weather measurement ratio for a specified change in the independent variable or exp(β) – 1 = percent change in GM air pollutant concentration or weather measurement for a specified change in the independent variable); [G] Includes coal and wood, gas and wood; [H] Using the Tukey method to adjust for multiple comparisons, p-values for tests of pairwise differences among primary fuels used for cooking were as follows: Gas only vs. Wood only: 0.41, Gas only vs. Other: 0.01, and Wood only vs. Other: 0.06; [I] Using the Tukey method to adjust for multiple comparisons, p-values for tests of pairwise differences among primary fuels used for cooking were as follows: Gas only vs. Wood only: 0.02, Gas only vs. Other: 0.01, and Wood only vs. Other: 0.57; [J] Includes lightbulb, line cable; [K] Includes candle and torch, fuel; [L] Using the Tukey method to adjust for multiple comparisons, p-values for tests of pairwise differences among types of non-electric light source in the home were as follows: Candle vs. Generator: <0.0001, Candle vs. Other or none: 1.00, and Generator vs. Other or none: < 0.0001; [M] Using the Tukey method to adjust for multiple comparisons, p-values for tests of pairwise differences among

types of non-electric light source in the home were as follows: Candle vs. Generator: 0.0003, Candle vs. Other or none: 1.00, and Generator vs. Other or none: 0.003.
